# Seleno-Functionalization of Quercetin Improves the Non-Covalent Inhibition of M^pro^ and Its Antiviral Activity in Cells against SARS-CoV-2

**DOI:** 10.3390/ijms22137048

**Published:** 2021-06-30

**Authors:** Francesca Mangiavacchi, Pawel Botwina, Elena Menichetti, Luana Bagnoli, Ornelio Rosati, Francesca Marini, Sérgio F. Fonseca, Laura Abenante, Diego Alves, Agnieszka Dabrowska, Anna Kula-Pacurar, David Ortega-Alarcon, Ana Jimenez-Alesanco, Laura Ceballos-Laita, Sonia Vega, Bruno Rizzuti, Olga Abian, Eder J. Lenardão, Adrian Velazquez-Campoy, Krzysztof Pyrc, Luca Sancineto, Claudio Santi

**Affiliations:** 1 Group of Catalysis, Synthesis and Organic Green Chemistry, Department of Pharmaceutical Sciences, University of Perugia Via del Liceo 1, 06100 Perugia, Italy; francesca.mangiavacchi@studenti.unipg.it (F.M.); elena.menichetti1@studenti.unipg.it (E.M.); luana.bagnoli@unipg.it (L.B.); ornelio.rosati@unipg.it (O.R.); francesca.marini@unipg.it (F.M.); 2 Virogenetics Laboratory of Virology, Malopolska Centre of Biotechnology, Jagiellonian University, Gronostajowa 7a, 30-387 Krakow, Poland; pawel.botwina@doctoral.uj.edu.pl (P.B.); agnieszka.dabrowska@doctoral.uj.edu.pl (A.D.); anna.kula-pacurar@uj.edu.pl (A.K.-P.); k.a.pyrc@uj.edu.pl (K.P.); 3 Microbiology Department, Faculty of Biochemistry, Biophysics and Biotechnology, Jagiellonian University, Gronostajowa 7, 30-387 Krakow, Poland; 4 School of Science and Technology, Chemistry Division, University of Camerino, Via S. Agostino, 62032 Camerino, Italy; 5 Laboratório de Síntese Orgânica Limpa—LASOL, CCQFA, Universidade Federal de Pelotas—UFPel, P.O. Box 354, 96010-900 Pelotas, Brazil; tec.sergio_fonseca@yahoo.com.br (S.F.F.); laura.abenante2018@gmail.com (L.A.); diego.alves@ufpel.edu.br (D.A.); lenardao@ufpel.edu.br (E.J.L.); 6 Institute for Biocomputation and Physics of Complex Systems (BIFI), Joint Units IQFR-CSIC-BIFI, and GBsC-CSIC-BIFI, Universidad de Zaragoza, 50018 Zaragoza, Spain; dortega@bifi.es (D.O.-A.); ajimenez@bifi.es (A.J.-A.); ceballos.laita@gmail.com (L.C.-L.); svega@bifi.es (S.V.) bruno.rizzuti@cnr.it (B.R.); oabifra@unizar.es (O.A.); adrianvc@unizar.es (A.V.-C.); 7 Departamento de Bioquímica y Biología Molecular y Celular, Universidad de Zaragoza, 50009 Zaragoza, Spain; 8 Instituto de Investigación Sanitaria de Aragón (IIS Aragon), 50009 Zaragoza, Spain; 9 CNR-NANOTEC, SS Rende (CS), Licryl-UOS Cosenza and CEMIF.Cal, Department of Physics, University of Calabria, 87036 Rende, Italy; 10 Instituto Aragonés de Ciencias de la Salud (IACS), 50009 Zaragoza, Spain; 11 Centro de Investigación Biomédica en Red en el Área Temática de Enfermedades Hepáticas Digestivas (CIBERehd), 28029 Madrid, Spain; 12 Fundación ARAID, Gobierno de Aragón, 50018 Zaragoza, Spain

**Keywords:** selenium, flavanols, main protease, SARS-CoV-2

## Abstract

The development of new antiviral drugs against SARS-CoV-2 is a valuable long-term strategy to protect the global population from the COVID-19 pandemic complementary to the vaccination. Considering this, the viral main protease (M^pro^) is among the most promising molecular targets in light of its importance during the viral replication cycle. The natural flavonoid quercetin **1** has been recently reported to be a potent M^pro^ inhibitor in vitro, and we explored the effect produced by the introduction of organoselenium functionalities in this scaffold. In particular, we report here a new synthetic method to prepare previously inaccessible C-8 seleno-quercetin derivatives. By screening a small library of flavonols and flavone derivatives, we observed that some compounds inhibit the protease activity in vitro. For the first time, we demonstrate that quercetin (**1**) and 8-(*p*-tolylselenyl)quercetin (**2d**) block SARS-CoV-2 replication in infected cells at non-toxic concentrations, with an IC_50_ of 192 μM and 8 μM, respectively. Based on docking experiments driven by experimental evidence, we propose a non-covalent mechanism for M^pro^ inhibition in which a hydrogen bond between the selenium atom and Gln189 residue in the catalytic pocket could explain the higher M^pro^ activity of **2d** and, as a result, its better antiviral profile.

## 1. Introduction

Flavonoids are valuable natural polyphenolic compounds endowed with a variety of biological properties [1] and are considered privileged scaffolds for rational drug design. This group of compounds has been extensively studied, primarily because of their pronounced antioxidant activity, which can proceed by several functioning mechanisms [1,2,3,4,5]. Glutathione peroxidases (GPx) are a family of phylogenetically related redox enzymes developed by living systems as protection against reactive oxygen species. Most of them are characterized by the presence of a selenocysteine in which the selenium atom is the actual catalyst for the glutathione-mediated reduction of peroxides [6], accounting for the essential role of selenium as a micronutrient in mammals [7]. A synergistic interaction between quercetin and its homologue catechin with GPx was proved by Watanabe and co-workers. The reversal of the hydrogen peroxide-induced cell damage mediated by such flavonoids could be observed only in selenium-supplemented, GPx-overexpressing cells, suggesting a direct interplay between flavonoids and the selenium-centered antioxidant system [8]. 

In 2020, a global pandemic caused by the SARS-CoV-2 spread worldwide [9], and while efforts were made to develop effective therapeutic strategies, none of the drugs included in clinical trials offered a significant benefit. Even if vaccination is the method of choice for viral infection containment, one must consider that some people will not develop appropriate immunity, and the disease will remain a cause of severe infections and fatal cases, especially in the high-risk groups. Furthermore, the emergence of escape variants will likely increase the number of affected individuals. Consequently, the development of small molecules able to block key steps of the viral replicative cycle is in high demand.

In this scenario, the seminal paper by Haitao Yang and co-workers, in which was reported the isolation of the SARS-CoV-2 main protease (M^pro^ or 3CL^pro^) and the identification of some of its inhibitors, is worth mentioning [10]. In their study, among other compounds, ebselen, one of the most investigated organoselenium compounds [11,12], was identified as the most potent M^pro^ inhibitor, with half-maximal inhibitory concentration (IC_50_) in the nanomolar range. Antiviral activity observed in cell models was noted at low micromolar levels. A few months later, some of us reported a fast in vitro screening meant to discover M^pro^ inhibitors and identified the flavonoid quercetin as a lead compound binding to the active site with an inhibition constant of 7.4 μM [13]. More recently, it was also demonstrated that rutin, a glycosylated conjugate of quercetin that commonly occurs as a natural flavonoid, shares the same properties as the parent compound, although with a slightly lower potency [14].

Considering our interest in the synthesis of organoselenium compounds with antioxidant activity, in light of the above-reported evidence and in the frame of our efforts to develop antimicrobial compounds [15,16,17,18], we sought to prepare novel quercetin derivatives decorated with organochalcogen moieties. These new hybrid compounds could present improved M^pro^ inhibition and be endowed with the ability to hamper SARS-CoV-2 replication in a cellular context, thus being suitable to be considered for preclinical studies required for the development of direct antiviral agents.

**Figure 1:**
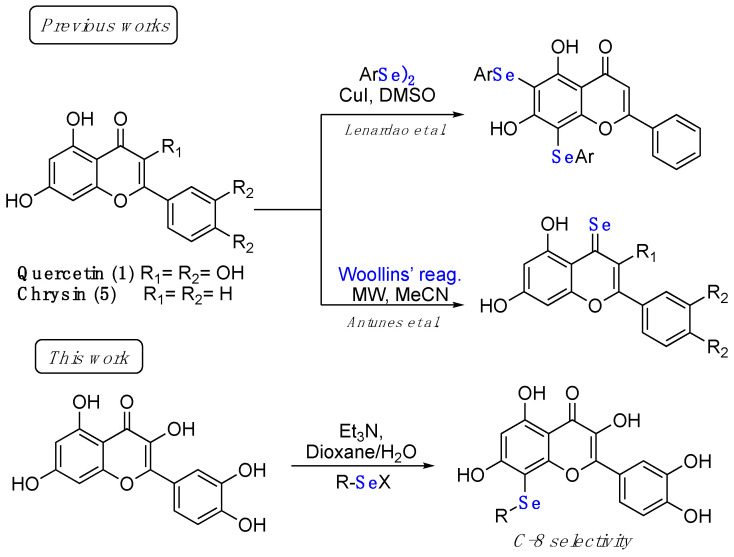
Examples of Se-functionalized flavonoids.

Some examples of flavonoids functionalized with selenium moieties have been previously reported in the literature and are collected in Figure 1. Double-selenenylated chrysin derivatives (at C-6 and C-8 positions) having an improved antioxidant and anticancer activity with respect to the parent natural compound were prepared by some of us through a CuI-catalyzed C–Se coupling reaction [19]. 

A different seleno-functionalization was proposed by Antunes et al. with the treatment of either quercetin or chrysin derivatives with Woollins’ reagent under microwave irradiation, and the consequent introduction of a selone functionality at the C-4 position. Moreover, in this case the introduction of a selenium atom in the flavonol scaffold resulted in increased radical scavenging and anticancer activities, the latter demonstrated in a panel of different cancer cell lines [20].

In this work, we report unprecedented reaction conditions for the selective functionalization of the quercetin scaffold at the C-8 position with an alkyl/aryl organoselenium moiety through a base-activated aromatic electrophilic substitution. All the new compounds were fully characterized and assayed for the ability to inhibit SARS-CoV-2 M^pro^ along with some previously prepared chrysin derivatives. The most potent derivatives were tested for their antiviral properties in a cellular context, identifying derivatives suitable for further preclinical evaluation. 

## 2. Results and Discussion

### 2.1. Synthesis 

In the frame of flavonoid modifications, some of us recently implemented a green protocol for the deuteration of quercetin 3-*O*-rutinoside (rutin) at the aromatic positions under basic conditions [21].

Using a similar strategy, we envisioned the possibility to exploit the reactivity of flavonols with electrophilic selenium reagents for the C–Se bond formation at the aromatic position of the resorcinol ring. Preliminary investigations (collected in Table 1) started from the screening of the base and the electrophile as the best partners in the electrophilic aromatic substitution. Based on the previously collected evidence [21], quercetin (**1**) and tris(hydroxymethyl)aminomethane (Tris), in a molar ratio of 1:5, were mixed and dissolved in the solvent (dioxane/water 4:1). Then, 2 molar equivalents of the electrophile were added, and the mixture was stirred at room temperature until the complete consumption of the starting material, monitored by TLC analysis. Among different selenium-centered electrophiles, only PhSeCl, PhSeOTf [22], and *N*-PhSe-phthalimide (NPSeP) afforded, at room temperature, the corresponding mono- and bis-selenenylated derivatives (**2a** and **3**, respectively), even if in poor to moderate yields (entries 1–3). Other electrophiles, such as PhSeBr or PhSeI (not shown in Table 1), afforded a complex mixture of unidentified compounds. A large amount of diphenyl diselenide (detected by TLC and in some cases isolated by chromatography) probably indicated that the nature of the anion has a role in the stability of the electrophile under the applied basic conditions and bromide and iodide derivatives are more prone to decomposition. Likely for the same reason, when the reactions were conducted at 80 °C (entries 4 and 5), slightly reduced yields resulted. To prevent the decomposition of the electrophile, the reactions with PhSeCl and NPSeP were repeated under an argon atmosphere, reaching moderate to good yields of compounds **2a** and **3** after chromatographic purification. Notably, the two different reagents showed a different chemoselectivity; PhSeCl gave the prevalent formation of the C-8 functionalized quercetin **2a** in the presence of traces of the double-functionalized compound **3**, both using 5 and 10 equivalents of base (entries 6 and 8). On the contrary, using NPSeP, **2a** and **3** were obtained in an overall yield of 81% but in a ratio of 37:63 (entry 7). Considering that the selective C-8 selenenylation of flavonoids is an unprecedented synthetic opportunity, other bases were investigated according to the conditions reported in Table 1 (entries 8–9), and triethylamine afforded the best results in the synthesis of **2a**, which was isolated in 55% yield (entry 9).

**Figure 2:**
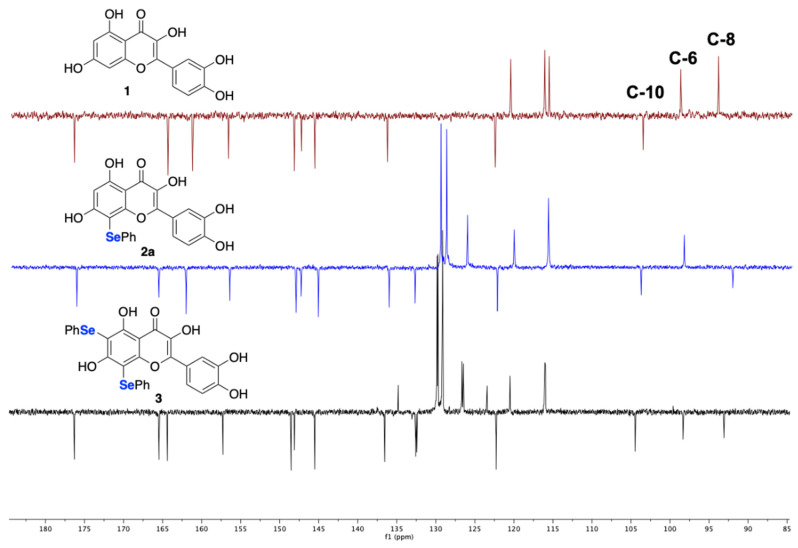
^13^C spectra of **1**, **2a**, and **3** recorded using the APT pulse sequence.

The structure of **2a** was assigned comparing the ^13^C NMR spectra of compounds **1**, **2a**, and **3** acquired using a standard APT sequence (attached proton test; Figure 2). Starting from the selected ^13^C NMR resonances unambiguously assigned in the literature [23] (spectrum in red color), the analysis clearly indicates that in **2a** C-8 is a quaternary carbon (spectrum in blue color) and that in **3** both C-6 and C-8 are quaternary (spectrum in black color).

Once the best conditions for the synthesis were identified, the scope of the reaction was explored using different alkyl and ary selenyl chlorides **5a**–**e**, which are commercially available (**5a**) or generated in situ (**5b**–**e**) by the treatment of the corresponding diselenides (**4b**–**e**) with a stoichiometric amount of SO_2_Cl_2_. Diselenides were prepared according to previously reported procedures [24,25,26], and the freshly prepared selenyl chlorides were immediately used after evaporation of the solvent and the unreacted sulfuryl chloride under reduced pressure.

The data referring to the synthesis of the monoselenenylated compounds (**2a**–**e**) are summarized in Table 2, and with the only exception of **3**, in all the other cases the double-functionalized derivatives were formed in traces and thus not isolated and characterized. 

Even if yields ranged from acceptable, when using PhSeCl (**5a**) and alkylselenyl chlorides **5b**–**c** (entries 1–3), to poor, when using less electrophilic aryl (**5d**–**e**) selenenylating reagents (entries 4–5), the protocol reported herein is interesting because, to the best of our knowledge, it provides for the first time the possibility to access this class of monoselenenylated flavonoids. To confirm the regioselectivity of the reaction, a NOESY experiment was carried out on the butyl derivative **2c**. An Overhauser effect was observed between the hydrogen atoms of the alkyl chain and the H2’ and H6’ of the catechol (see Supplementary Materials), in agreement with the structure assigned based on the ^13^C NMR analysis.

### 2.2. Evaluation of M^pro^ Inhibition and Antiviral Assay

**Figure 3:**
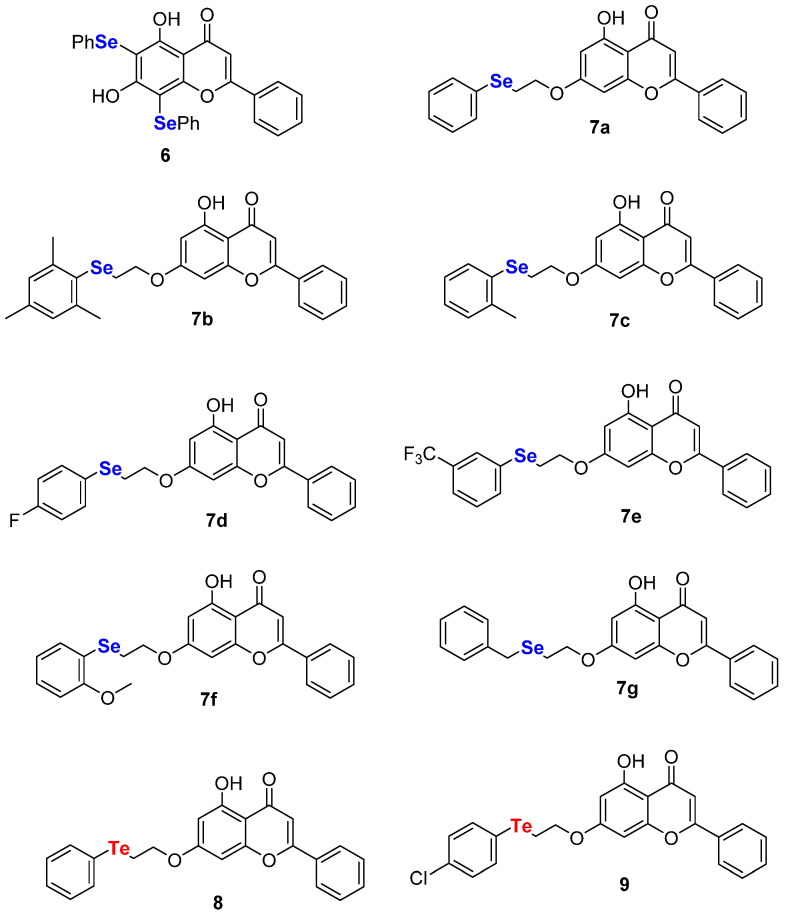
Chalcogen-containing chrysin derivatives.

The small compound library herein synthesized, together with a series of selenium- (**6**, **7a**–**g**) and tellurium-containing (**8**, **9**) chrysin analogues [27], reported in Figure 3, was assayed through in vitro screening based on M^pro^ hydrolytic activity using a Förster resonance energy transfer (FRET) substrate, very recently implemented by some of us [13]. 

**Figure 4:**
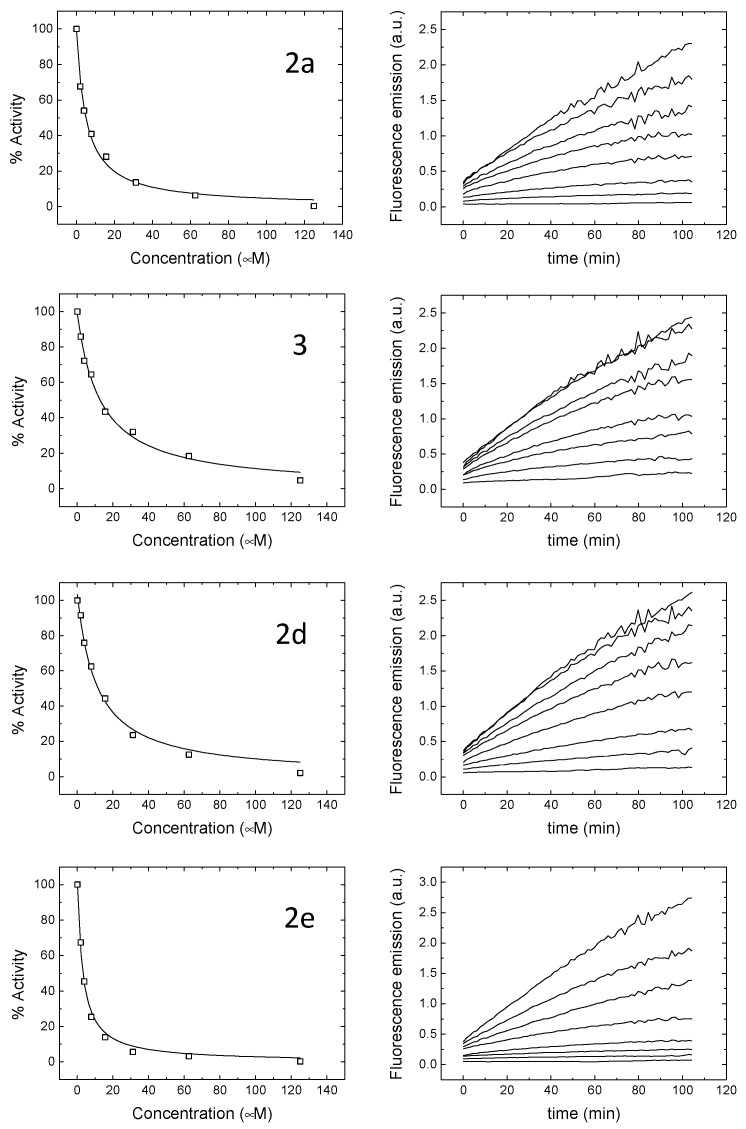
Inhibition curves (**left**) and time-course monitoring of M^pro^ activity at increasing concentrations of compounds (**right**). Increasing the concentration of compounds results in diminished M^pro^ activity. Nonlinear least-square regression data analysis was employed to determine the inhibition constant (K_i_) and the half-maximal inhibitory concentration (IC_50_).

As a first line of screening, all the compounds were assayed at 100 μM. Hits consisted of compounds diminishing the M^pro^ enzymatic activity and were then investigated in depth to determine their inhibition constant (*K*_i_) and their half-maximal inhibitory concentration (IC_50_). The obtained values are collected in Table 3 and Figure 4 and compared to those reported in the literature for quercetin (**1**), which was selected as the reference compound. 

As a general comment, it is possible to underline that the introduction of an organoselenyl moiety in the quercetin structure endowed the molecule with a good ability to inhibit M^pro^. Indeed, quercetin has a *K*_i_ of 7.4 μM, whereas the vast majority of the tested compounds displayed a higher potency, with the sole exception of **6** and **2b**, which were devoid of any noticeable anti-M^pro^ inhibitory activity. The selective introduction of the phenylselenyl moiety at the C-8 position was highly effective, while the double substitution resulted in a drop in the activity (**2a** vs. **3**), even if **3** is still a better M^pro^ inhibitor when compared to quercetin **1**. The quercetin scaffold seems essential, as was demonstrated by the fact that the 5,7-dihydroxy analogue chrysin derivative (**6**) was completely inactive, indicating that the higher degree of hydroxylation improves the target recognition. The best-in-class compound, which surely deserves further investigation, is **2e**, with a *p*-methoxyphenylselenyl substituent at the C-8 position. Aliphatic substituents at the selenium atom are not well tolerated, since the butyl (**2c**) and decyl (**2b**) derivatives yielded molecules with a poorer propensity to block the enzymatic activity of M^pro^. 

Many of the inhibitors identified and under development interact covalently with M^pro^. We assessed the reversibility of the interaction of compounds **1** and **2d** by performing experiments aimed at evaluating whether or not the enzyme–inhibitor complex dissociates under equilibrium: dialysis and size exclusion chromatography followed by enzymatic activity quantification and near-UV circular dichroism spectra (inhibitor-bound and inhibitor-free ones display markedly different near-UV circular dichroism spectra). In the case of M^pro^, the far-UV circular dichroism spectrum is quite insensitive to ligand binding, but the near-UV circular dichroism spectrum changes considerably [13]; therefore, enzymatic activity and spectral properties can be employed as specific signature for ligand-free M^pro^. Both compound **1** and compound **2d** exhibited a reversible interaction with SARS-CoV-2 M^pro^, provided that dilution is enough to dissociate both compounds from M^pro^ (see Supplementary Materials), confirming the non-covalent interaction of the selenium-derived compound.

Surprisingly, tellurium derivatives displayed a potent anti-M^pro^ activity (**9** and **8**), better than that displayed by the close selenium analogue, **7a**, and by the whole set of compounds. Of course, there are several concerns about the toxicity of organotellurium compounds [28], which were indirectly confirmed by the in cellulo tests (reported below).

**Figure 5:**
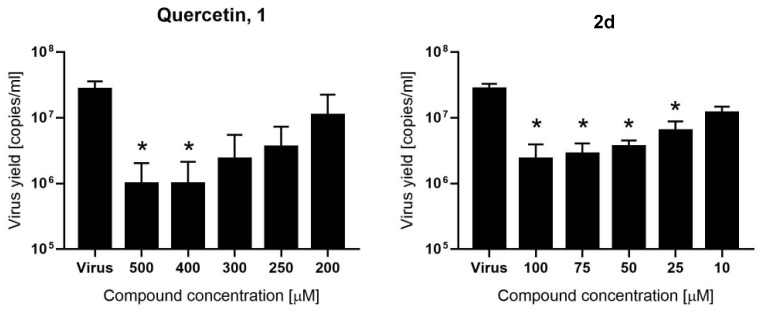
Inhibition of SARS-CoV-2 replication by quercetin (compound **1**) and compound **2d**. Virus replication was evaluated at the given compound concentrations using RT-qPCR and the data are presented as SARS-CoV-2 RNA copies per mL of the original sample. Bars show mean value ± SEM from three independent experiments. The significance level (* *p* < 0.05) is denoted with an asterisk in the graph.

The most promising M^pro^ inhibitors (**1**, **2a**, **2d**, **2e**, **3**, **8**, and **9**) were successively assayed in an in vitro model of SARS-CoV-2 infection. The ability of each compound to inhibit the virus replication was evaluated by infecting confluent Vero monolayers with SARS-CoV-2 in the presence of a given concentration of each compound or DMSO as a control. Mock-infected cells were also used as a negative control. In the initial experiments all compounds were tested at five concentrations (100, 75, 50, 25, and 10 µM). After incubation, the cells were scored under the inverted light microscope for the presence or absence of the SARS-CoV-2-related cytopathic effect (CPE) and cytotoxicity (Table S1). Two compounds inhibited the development of the CPE (**2a** and **2d**). No CPE reduction was recorded for other compounds (**2e**, **3**, **8**, and **9**) at non-toxic concentrations. Compounds **8** and **9** precipitated and were toxic, making it impossible to interpret the CPE reduction for compound **8**. The two most promising compounds (**2a** and **2d**) and compound **1**, tested at higher concentrations (500, 400, 300, 250, and 200 μM), were further evaluated for the inhibition of the SARS-CoV-2 replication by means of RT-qPCR analysis. Analysis of **2a** did not indicate any inhibition of SARS-CoV-2 (Figure S1). The cytotoxicity of quercetin (500–100 µM) and **2d** compound (100–10 µM) was also evaluated on Vero cells and none of the tested concentrations exhibited cytotoxicity (Figure S2). The same dose ranges of compounds were also tested in the in vitro cellular SARS-CoV-2 infection model (Tables S1 and S2) and morphological changes were observed for **2d** at 100 µM concentration (Table S1). Both compounds exhibited CPE reduction as observed under the inverted light microscope (Figure S3). Importantly, our RT-qPCR analysis, shown in Figure 5, revealed that quercetin significantly inhibited SARS-CoV-2 infection at relatively high concentrations, with IC_50_ = 192 µM (Figure S4). Quercetin derivative **2d** inhibited the virus replication at noticeably lower concentration (IC_50_ = 8 µM, Figure S4).

This finding is in line with the M^pro^ inhibition, where **2d** is among the most potent compounds. No inhibition was observed for compounds **2e** and **3**, at non-toxic concentrations, although these compounds are able to hamper the M^pro^ activity. The reason behind the lack of activity for these compounds could be their poor bioavailability/penetration into the cellular compartment, where the virus replicates, or their toxicity.

### 2.3. Molecular Docking of the Representative Compound ***2d*** to SARS-CoV-2 M^pro^

**Figure 6:**
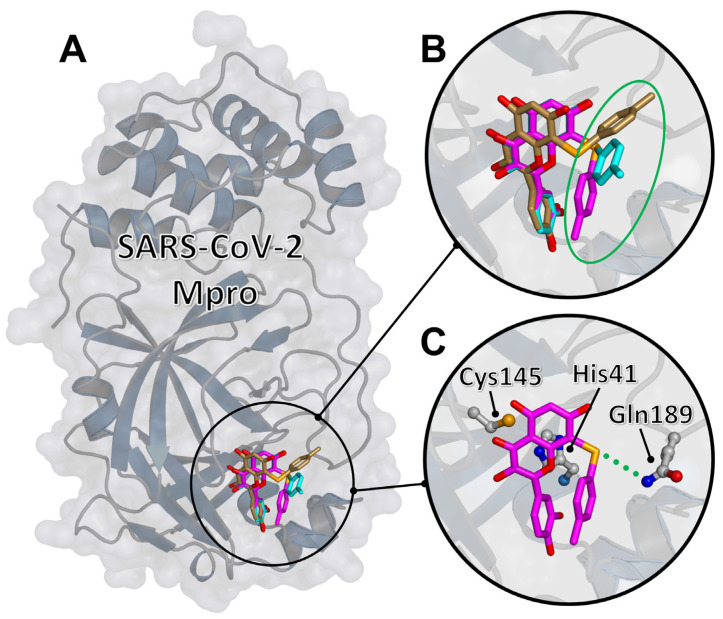
Molecular docking of compound **2d** to SARS-CoV-2 M^pro^. (**A**) The best three docking poses bound in the protein active site. (**B**) Detail of the docking poses highlighting the quercetin scaffold approximately superimposed, while the chemical adduct (circled in green) shows a large variability. (**C**) The most favorable docking pose (binding energy −8.2 kcal/mol), with the side chains of selected protein residues (the catalytic dyad His41/Cys145 and the hydrogen bonded residue Gln189) explicitly shown.

The binding to M^pro^ of the most promising compound found in the experiment, compound **2d**, was modeled in silico by using molecular docking simulations, largely following a protocol that some of us have used to study the binding of quercetin and its analogue rutin [13,14]. A blind search on the entire protein surface was performed at very high exhaustiveness, and using two reference crystallographic structures [29]. The results shown in Figure 6 indicate that compound **2d** was predicted to bind in the protein active site, with the double ring of the quercetin moiety in direct interaction with the catalytic dyad, including residues His41 and Cys145. The best docking poses, assessed in terms of both the most favorable affinity score and the number of binding modes in the same docking cluster, shared a similar conformation. In particular, (i) the quercetin scaffold occupied a single well-defined position, in contact with the two catalytic residues (minimum distance: 3.5 and 3.7 Å with Cys145 and His41, respectively); (ii) the selenium atom was an acceptor of a hydrogen bond with the side chain of Gln189 (donor–acceptor distance: 3.8 Å); (iii) the adduct of quercetin had a larger variability in its position, having the possibility of forming a number of weak hydrophobic contacts with various chemical groups of the protein. The binding energy of the best three poses was –8.0 ± 0.2 kcal/mol, indicating a very favorable binding affinity.

A number of conclusions can be drawn from these observations. First, the quercetin scaffold is crucial to bind M^pro^, and has a distinct association mode to the protein active site. However, compared to the binding of (unmodified) quercetin [13], for compound **2d** the presence of a chemical adduct breaks the pseudo-symmetry in the molecular structure, reducing the possible anchoring modes of the parent scaffold to a single conformation. This is also at variance with rutin, whose chemical adduct still allows two possible binding modes for the sugar moiety [14]. The reason for this behavior is the presence of the selenium atom, which contributes to the binding of the compound by forming a hydrogen bond. We note that the presence of this conserved bond, together with the specificities of the nature of selenium as a hydrogen bond acceptor compared to other elements [30], would also explain in a rather straightforward way the strong potency of our quercetin derivatives with respect to the parent compound. In our simulations, compared to quercetin [13], not only compound **2d** showed a higher selectivity in the binding conformation, but the predicted affinity was also more favorable by 1 kcal/mol. The chemical adduct of compound **2d** had a higher variability in the conformation compared to the quercetin scaffold and contributed to a lesser extent to the binding. Therefore, we suggest that the binding of the other selenium-derivative compounds is modulated by subtle differences in the interactions of their adducts with the entrance region of the M^pro^ active site, and likely also by entropic contributions. These effects are difficult to capture by molecular docking due to the limitation of this technique, which does not take into account the dynamics of the protein–ligand complex.

## 3. Materials and Methods

### 3.1. Synthesis: General Remarks

Solvents and reagents were used as received unless otherwise noted. The starting materials are commercially available; diselenides **4b**–**f** [25,26] and chrysin derivatives **6**, **7a**–**g**, **8**, and **9** belong to the same batch described in ref. [27] and were synthesized as reported in the literature; the physical and spectral data of **6**, **7a**–**g**, **8**, and **9** are reported below. Reactions were conducted in round-bottom flasks and were stirred with Teflon-coated magnetic stirring bars. 

The analytical thin layer chromatography (TLC) was performed on silica gel 60 F254-precoated aluminum foil sheets (Merck, Darmstadt, Germany) and visualized by UV irradiation (Spectroline^®^ UV light, Sigma-Aldrich, St. Louis, MI, USA) or by using I_2_ or a KMnO_4_ stain (Merck, Darmstadt, Germany). Silica gel Kiesinger 60 (70–230 mesh) was used for flash column chromatography and a 240–400 mesh for normal column chromatography. NMR spectroscopic (Bruker, Fällanden, Switzerland) experiments were performed at 25 °C on a Bruker DRX 400 MHz (UniPG-Italy) and a Varian INOVA 300 MHz (UFRGS-Brazil).

^1^H and ^13^C NMR chemical shifts (δ) are reported in parts per million (ppm), and they are relative to TMS (0.0 ppm), and the residual solvent peak (DMSO-*d6*, 2.50 ppm or CDCl_3_, 7.27 for ^1^H NMR, and 39.52 ppm or 77.0 ppm for ^13^C NMR). ^77^Se chemical shifts (δ) are reported in parts per million (ppm), and they are relative to diphenyl diselenide (447 ppm) in DMSO-*d6*. Data are reported as follows: chemical shift (multiplicity, coupling constants, where applicable, and the number of hydrogen atoms). Abbreviations are as follows: s (singlet), d (doublet), t (triplet), q (quartet), dd (doublet of doublet), dt (doublet of triplet), tt (triplet of triplet), m (multiplet), br.s. (broad signal). Coupling constant (*J*) is quoted in Hz to the nearest 0.1 Hz. High-resolution mass spectrometry (HRMS) measurements were performed using a Synapt G2-Si mass spectrometer (Waters, Milford, USA) equipped with an APCI source and quadrupole time-of-flight mass analyzer. The mass spectrometer was operated in the positive and negative ion detection modes with discharge current set at 4.0 μA. The heated capillary temperature was 350 °C. The results of the measurements were processed using MassLynx 4.1 software (Waters, Milford, USA) incorporated into the instrument. Melting points (m.p.) were determined on Mel-Temp® apparatus or a Marte PFD III instrument (Brazil) with a 0.1 °C precision, and are uncorrected.

#### 3.1.1. General Procedure for the In Situ Formation of RSeCl (**5b**–**f**)

Diselenide **4b**–**f** (0.22 mmol) was placed in a round-bottom flask under an argon atmosphere, and dichloromethane (1 mL) was added. The resulting solution was stirred at 0 °C, and sulfuryl chloride (0.22 mmol) was added dropwise. The reaction mixture was stirred at room temperature for 1 h, while protected from light. After 1 h, the solvent was removed under reduced pressure, and the corresponding organoselenyl chloride was used without further purification.

#### 3.1.2. General Procedure for the Seleno-Functionalization of **1**

Quercetin **1** (0.2 mmol) and triethylamine (1.0 mmol) were placed in a round-bottom flask and sonicated until homogeneity. The flask was connected to a vacuum line, and several evacuations and purges with argon were made. Then, a mixture of 1,4-dioxane (1.5 mL) and water (0.5 mL) was added to solubilize the reagents, followed by the addition of RSeX (0.44 mmol) in 1,4-dioxane (0.5 mL). The reaction mixture was stirred for the time indicated in Table 2. The reactions were monitored by TLC (eluent CH_2_Cl_2_/MeOH 9:1). The reaction was quenched with 10% aqueous HCl, extracted with EtOAc (3 × 20 mL), dried over Na_2_SO_4_ and concentrated under reduced pressure. Products were purified by column chromatography using a mixture CH_2_Cl_2_/MeOH as eluent.

#### 3.1.3. Physical Data

**2-(3,4-Dihydroxyphenyl)-3,5,7-trihydroxy-8-(phenylselanyl)-4*H*-chromen-4-one (2a)** Yellow solid (m.p. 211–213 °C; 53 mg, 0.12 mmol, isolated yield 58% purified by silica gel chromatography; eluent DCM/MeOH 98:2). ^1^H NMR (400 MHz, DMSO-d6): δ 12.88 (s, 1H), 11.3 (br.s., 1H), 9.59 (s, 2H), 9.25 (s, 1H), 7.80–7.77 (m, 1H), 7.45–7.40 (m, 1H), 7.23–7.10 (m, 5H), 6.78 (d, *J* = 8.4 Hz, 1H), 6.43 (s, 1H) ppm. ^13^C NMR (100.62 MHz, DMSO-d6): δ 176.0, 165.5, 162.0, 156.4, 147.9, 147.3, 145.1, 136.0, 132.7, 129.4 (2C), 128.6 (2C), 126.0, 122.1, 120.0, 115.6, 115.5, 103.7, 98.2, 92.0 ppm. ^77^Se NMR (76.27 MHz, DMSO-d6): δ 222.0 ppm. HRMS (ESI-TOF): calc. for C_21_H_15_O_7_Se [M+H]^+^: 458.9983; found 458.9985.

**2-(3,4-Dihydroxyphenyl)-3,5,7-trihydroxy-8-(decylselenyl)-4*H*-chromen-4-one (2b)** Yellow solid (m.p. 155–159 °C; 50 mg, 0.10 mmol, isolated yield 48% purified by silica gel chromatography; eluent DCM/MeOH 98:2). ^1^H NMR (400 MHz, DMSO-d6): δ 12.73 (s, 1H), 9.54 (br.s, 3H), 7.90 (s, 1H), 7.74 (d, *J* = 7.5 Hz, 1H), 6.89 (d, *J* = 7.8 Hz, 1H), 6.35 (s, 1H), 2.80–2.77 (m, 2H), 1.44–1.03 (m, 16H), 0.82–0.79 (m, 3H) ppm. ^13^C NMR (100.62 MHz, DMSO-d6): δ 176.0, 165.0, 160.9, 156.3, 147.9, 147.2, 145.2, 135.8, 122.4, 120.0, 115.6, 115.5, 103.5, 97.9, 91.8, 31.3, 29.6, 29.0, 29.0, 28.8, 28.7, 28.5, 26.8, 22.2, 14.0 ppm. ^77^Se NMR (76.27 MHz, DMSO-d6): δ 115.7 ppm. HRMS (ESI-TOF): calc. for C_25_H_31_O_7_Se [M+H]^+^: 523.1235, found 523.1233.

**2-(3,4-Dihydroxyphenyl)-3,5,7-trihydroxy-8-(butylselenyl)-4*H*-chromen-4-one (2c)** Yellow solid (m.p. 159–163 °C; 40 mg, 0.09 mmol, isolated yield 46% purified by silica gel chromatography; eluent DCM/MeOH 98:2). ^1^H NMR (400 MHz, DMSO-*d6*): δ 12.72 (s, 1H), 9.73–9.32 (m, 3H), 7.88 (d, *J* = 2.1 Hz, 1H), 7.73 (dd, *J* = 2.0 and *J* = 8.5 Hz, 1H), 6.90 (d, *J* = 8.5 Hz, 1H), 6.35 (s, 1H), 2.79 (t, *J* = 7.2 Hz, 2H), 1.47–1.42 (m, 2H), 1.34–1.28 (m, 2H), 0.74 (t, *J* = 7.3 Hz, 3H) ppm. ^13^C NMR (100.62 MHz, DMSO-*d6*): δ 176.1, 165.1, 160.9, 156.3, 148.0, 147.2, 145.2, 135.8, 122.4, 120.1, 115.7, 115.5, 103.5, 97.9, 92.0, 32.0, 26.7, 22.1, 13.5 ppm. ^77^Se NMR (76.27 MHz, DMSO-*d6*): δ 115.4 ppm. HRMS (ESI-TOF): calc. for C_19_H_19_O_7_Se [M+H]^+^: 439.0296, found 439.0299. 

**2-(3,4-Dihydroxyphenyl)-3,5,7-trihydroxy-8-(p-tolylselanyl)-4*H*-chromen-4-one (2d)** Yellow solid (m.p. 203–206 °C; 40 mg, 0.06 mmol, isolated yield 32% purified by silica gel chromatography; eluent DCM/MeOH 98:2). ^1^H NMR (400 MHz, DMSO-*d6*): δ 12.85 (s, 1H), 9.67–9.59 (m, 2H), 9.27 (br.s, 1H), 7.80 (d, *J* = 2.1 Hz, 1H), 7.46 (dd, *J* = 2.1 Hz and *J* = 8.5 Hz, 1H), 7.13 (d, *J* = 8.1 Hz, 2H), 6.99 (d, *J* = 8.0 Hz, 2H), 6.79 (d, *J* = 8.5 Hz, 1H), 6.41 (s, 1H), 2.16 (s, 3H) ppm. ^13^C NMR (100.62 MHz, DMSO-*d6*): δ 176.0, 165.3, 161.9, 156.3, 147.9, 147.3, 145.1, 136.0, 135.4, 130.0 (2C), 129.1 (2C), 128.8, 122.2, 120.0, 115.6, 103.7, 98.1, 92.5, 20.6 ppm. ^77^Se NMR (76.27 MHz, DMSO-*d6*): δ 215.4 ppm. HRMS (ESI-TOF): calc. for C_22_H_17_O_7_Se [M+H]^+^ 473.0140, found 473.0140.

**2-(3,4-Dihydroxyphenyl)-3,5,7-trihydroxy-8-((4-methoxyphenyl)selanyl)-4*H*-chromen-4-one (2e)** Yellow solid (m.p. 194–197 °C; 20 mg, 0.04 mmol, isolated yield 21% purified by silica gel chromatography; eluent DCM/MeOH 98:2). ^1^H NMR (400 MHz, DMSO-*d6*): δ 12.84 (s, 1H), 9.60–9.33 (br.s, 3H), 7.85 (s, 1H), 7.54 (d, *J* = 7.7 Hz, 1H), 7.25 (d, *J* = 8.1 Hz, 2H), 6.85 (d, *J* = 8.2 Hz, 1H) 6.78 (d, *J* = 8.1 Hz, 2H), 6.40 (s, 1H), 3.64 (s, 3H) ppm. ^13^C NMR (100.62 MHz, DMSO-*d6*): δ 176.0, 165.3, 161.8, 158.3, 156.2, 148.0, 147.3, 145.2, 136.0, 131.6 (2C), 122.2 (2C), 120.1, 115.7, 115.6, 115.1 (2C), 103.6, 98.1, 93.5, 55.2 ppm. ^77^Se NMR (76.27 MHz, DMSO-*d6*): δ 208.1 ppm. HRMS (ESI-TOF): calc. for C_22_H_17_O_8_Se [M+H]^+^: 489.0089 found 489.0092.

**2-(3,4-Dihydroxyphenyl)-3,5,7-trihydroxy-6,8-bis(phenylselanyl)-4*H*-chromen-4-one (3)** Yellow solid (m.p. 206–210 °C; 62.5 mg, 0.10 mmol, isolated yield 51% purified by silica gel chromatography; eluent DCM/MeOH 98:2). ^1^H NMR (400 MHz, DMSO-*d6*): δ 13.93 (s, 1H), 10.34 (s, 1H), 9.81 (s, 1H), 9.71 (s, 1H), 9.31 (s, 1H), 7.83 (s, 1H), 7.51–7.49 (m, 1H), 7.27–7.16 (m, 10H), 6.81 (d, *J* = 8.5 Hz, 1H) ppm. ^13^C NMR (100.62 MHz, DMSO-*d6*): δ 175.8, 165.1, 163.9, 156.9, 148.1, 147.7, 145.1, 136.1, 134.4, 132.2, 132.0, 129.4 (2C), 129.3 (2C), 128.7 (3C), 126.2, 126.0, 123.0, 121.9, 120.1, 115.6, 104.0, 98.0, 92.7 ppm. ^77^Se NMR (76.27 MHz, DMSO-*d6*): δ 228.6, 226.0 ppm. HRMS (ESI-TOF): calc. for C_27_H_19_O_7_Se_2_ [M+H]^+^: 614.9461, found 614.9456.

**5,7-Dihydroxy-2-phenyl-6,8-bis(phenylselanyl)-4*H*-chromen-4-one (6)** [19] Yellow solid (m.p. 188.7–190.6 °C; 251 mg, 0.44 mmol, isolated yield 89% purified by silica gel chromatography; eluent hexane/AcOEt 80:20). ^1^H NMR (400 MHz, CDCl_3_/DMSO-*d*_6_): δ 14.08 (s, 1H); 7.93 (s, 1H); 7.84 (d, *J* = 7.0 Hz, 2H), 7.52–7.38 (m, 2H), 7.33–7.31 (m, 1H); 7.20–7.15 (m, 3H); 6.72 9s, 1H) ppm. ^13^C NMR (100 MHz, CDCl_3_/DMSO-*d*_6_): δ 180.6, 163.8, 163.2, 162.3, 187.1, 130.5, 130.3, 130.0, 128.9, 127.7, 127.5, 127.4, 127.3, 124.9, 124.7, 124.6, 103.8, 103.7, 97.8, 91.7 ppm. 

**5-Hydroxy-2-phenyl-7-(2-(phenylselanyl)ethoxy)-4*H*-chromen-4-one (7a)** [27] White solid (m.p. 155–156 °C; 377 mg, 0.86 mmol, isolated yield 86% purified by silica gel chromatography; eluent hexane/AcOEt 20:80). ^1^H NMR (300 MHz, CDCl_3_): δ 12.69 (s, 1H); 7.86–7.83 (m, 2H); 7.59–7.50 (m, 5H); 7.31–7.29 (m, 3H); 6.63 (s, 1H); 6.38 (d, *J* = 7.2 Hz, 1H); 6.26 (d, *J* = 2.1 Hz, 1H); 4.24 (t, *J* = 7.2 Hz, 2H); 3.23 (t, *J* = 7.2 Hz, 2H) ppm. ^13^C NMR (75 MHz, CDCl_3_): δ 182.3, 164.2, 163.8, 162.0, 157.6, 133.2, 131.8, 131.1, 129.2, 129.0, 127.5, 126.2, 105.7, 98.5, 92.9, 25.5 ppm.

**5-Hydroxy-7-[(2-mesitylselanyl)ethoxy]-2-phenyl-4*H*-chromen-4-one (7b)** [27] White/pink solid (m.p. 135.7–138.4°C; 289 mg, 0.60 mmol, isolated yield 60% purified by silica gel chromatography; eluent hexane/AcOEt 20:80). ^1^H NMR (300 MHz, CDCl_3_): δ 12,70 (s, 1H); 7.86–7.84 (m, 2H); 7.53–7.52 (m, 3H); 6.95 (s, 2H); 6.63 (s, 1H); 6.35 (d, *J* = 2.2 Hz, 1H); 6.23 (d, *J* = 2.2. Hz, 1H); 4.12 (t, *J* = 7.0 Hz, 2H); 2.99 9t, *J* = 7.0 Hz, 2H); 2.55 (s, 6H); 2.26 (s, 3H) ppm. ^13^C NMR (75 MHz, CDCl_3_): δ 182.3, 164.9, 164.2, 163.9, 162.1, 157.6, 143.1, 138.6, 131.8, 131.2, 129.0, 128.6, 126.4, 126.2, 105.8, 105.7, 98.4, 93.0, 77.4, 76.6, 67.9, 25.2, 24.5, 20.9 ppm.

**5-Hydroxy-2-phenyl-7-[2-(o-tolylselanyl)ethoxy]-4*H*-chromen-4-one (7c)** [27] Light yellow solid (m.p. 123.9–127.4 °C; 321 mg, 0.73 mmol, isolated yield 71% purified by silica gel chromatography; eluent hexane/AcOEt 20:80). ^1^H NMR (300 MHz, CDCl_3_): δ 12.70 (s, 1H); 7.87–7.84 (m, 2H); 7.53–7.50 (m, 4H); 7.26–7.10 (m, 3H); 6.64 (s, 1H); 6.40 (d, *J* = 2.2 Hz, 1H); 6.28 40 (d, *J* = 2.2 Hz, 1H); 4.23 (t, *J* = 7.2 Hz, 2H); 3.21 (t, J = 7.2 Hz, 2H); 2.45 (s, 3H) ppm. ^13^C NMR (75 MHz, CDCl_3_): δ 182.4, 164.2, 163.9, 162.1, 157.6, 139.9, 132.3, 131.8, 131.2, 130.2, 129.8, 129.0, 127.4, 126.7, 126.2, 105.8, 98.6, 93.0, 67.7, 24.4, 22.5 ppm.

**7-{2-[(4-Fluorophenyl)selanyl]ethoxy}-5-hydroxy-2-phenyl-4*H*-chromen-4-one (7d)** [27] White solid (m.p. 171.1–173.7 °C; 282 mg, 0.62 mmol, isolated yield 62% purified by silica gel chromatography; eluent hexane/AcOEt 20:80). ^1^H NMR (300 MHz, CDCl_3_): δ 12.70 (s, 1H); 7.87–7.84 (m, 2H); 7.59–7.53 (m, 5H); 7.07–6.97 (m, 2H); 6.64 (s, 1H); 6.36 (d, *J* = 2.2 Hz, 1H); 6.26 (d, *J* = 2.2 Hz, 1H); 4.23 (t, *J* = 7.1 Hz, 2H); 3.18 (t, *J* = 7.1 Hz, 2H) ppm. ^13^C NMR (75 MHz, CDCl_3_): δ 182.4, 164.3, 163.9, 162.6 (d, ^1^*J*_C-F_ = 247.9 Hz), 162.1, 157.6, 136.0 (d, ^3^*J*_C-F_ = 8.0 Hz), 131.8, 131.1 129.0, 126.2, 123.2 (d, ^4^*J*_C-F_ = 3.4 Hz), 116.4 (d, ^2^*J*_C-F_ = 21.5 Hz), 105.8, 98.5, 92.9, 67.8, 26.4 ppm.

**5-Hydroxy-2-phenyl-7-{2-[(3-(trifluoromethyl)phenyl]selanyl)ethoxy}-4*H*-chromen-4-one (7e)** [27] White solid (m.p. 154.1–157.7 °C; 289 mg, 0.57 mmol, isolated yield 57% purified by silica gel chromatography; eluent hexane/AcOEt 20:80). ^1^H NMR (300 MHz, CDCl_3_): δ 12.70 (s, 1H); 7.87–7.44 (m, 3H); 7.74 (d, *J* = 7.7 Hz, 1H); 7.55–7.50 (m, 4H); 7.41 (t, *J* = 7.7 Hz, 1H); 6.64 (s, 1H); 6.39 (d, *J* = 2.2 Hz, 1H); 6.27 (d, *J* = 2.2. Hz, 1H); 4.29 (t, *J* = 6.8 Hz, 2H); 3.30 (t, *J* = 6.8 Hz, 2H) ppm^13^C NMR (75 MHz, CDCl_3_): δ 182.4, 164.9, 164.0, 163.9, 162.1, 157.6, 136.0 (q, ^4^*J*_C-F_ = 1.26 Hz), 131.8, 131.4 (q, ^2^*J*_C-F_ = 32.5 Hz), 131.1, 130.4, 129.5 (q, ^3^*J*_C-F_ = 3.80 Hz), 129.0, 126.2, 124.1 (q, ^3^*J*_C-F_ = 3.77 Hz), 123.5 (q, ^1^*J*_C-F_ = 272.7 Hz), 105.8, 105.7, 98.4, 93.0, 67.8, 25.9 ppm. 

**5-Hydroxy-7-{2-[(2-methoxyphenyl)selanyl]ethoxy}-2-phenyl-4*H*-chromen-4-one (7f)** [27] Grey solid (m.p. 132.9–135.6 °C; 378 mg, 0.84 mmol, isolated yield 81% purified by silica gel chromatography; eluent hexane/AcOEt 20:80). ^1^H NMR (300 MHz, CDCl_3_): δ 12.65 (s, 1H); 7.85–7.82 (m, 2H); 7.51–7.44 (m, 4H); 7.25 (ddd, *J* = 8.1, 7.4 and 1.6 Hz, 1H); 6.91–6.85 (m, 2H); 6.61 (s, 1H); 6.40 (d, *J* = 2.2 Hz, 1H); 6.27 (d, *J* = 2.2 Hz, 1H); 4.26 (t, *J* = 7.3 Hz, 2H); 3.88 (s, 3H); 3.23 (t, *J* = 7.3 Hz, 2H) ppm. ^13^C NMR (75 MHz, CDCl_3_): δ 182.4, 164.9, 164.4, 163.9, 162.2, 158.3, 157.7, 132.4, 131.7, 131.3, 129.0, 128.6, 126.2, 121.5, 117.9, 110.8, 105.8, 105.8, 98.6, 93.1, 68.2, 55.8, 23.0 ppm.

**7-[2-(Benzylselanyl)ethoxy]-5-hydroxy-2-phenyl-4*H*-chromen-4-one (7g)** [27] Green solid (m.p. 115.4- 118.2 °C; 336 mg, 0.74 mmol, isolated yield 74% purified by silica gel chromatography; eluent hexane/AcOEt 20:80). ^1^H NMR (300 MHz, CDCl_3_): δ 12.71 (s, 1H); 7.87–7.84 (m, 2H); 7.52–7.50 (m, 3H); 7.32–7.25 (m, 5H); 6.63 (s, 1H); 6.41 (d, *J* = 6.7 Hz, 1H); 6.28 (d, *J* = 2.2 Hz, 1H); 4.13 (t, *J* = 7.0 Hz, 2H); 3.89 (t, *J* = 6.7 Hz, 2H) ppm. ^13^C NMR (75 MHz, CDCl_3_): δ 182.3, 164.2, 163.8, 162.1, 157.6, 138.8, 131.8, 131.1, 129.0, 128.8, 128.6, 126.9, 126.2, 105.7, 105.7, 98.5, 93.0, 68.7, 27.7, 21.4 ppm.

**5-Hydroxy-2-phenyl-7-[2-(phenyltellanyl)ethoxy]-4*H*-chromen-4-one (8)** [27] Yellow solid (m.p. 128–129 °C; 407 mg, 0.82 mmol, isolated yield 87% purified by silica gel chromatography; eluent hexane/AcOEt 20:80). ^1^H NMR (300 MHz, CDCl_3_): δ 12.69 (s, 1H); 7.86–7.79 (m, 4H); 7.53–7.50 (m, 3H); 7.33–7.21 (m, 3H); 6.63 (s, 1H); 6.37 (d, *J* = 2.2 Hz, 1H); 6.26 (d, *J* = 2.2 Hz, 1H); 4.34 (t, *J* = 7.6 Hz, 2H); 3.19 (t, *J* = 7.6 Hz, 2H) ppm. ^13^C NMR (75 MHz, CDCl_3_): δ 182.3, 164.1, 163.8, 162.0, 157.7, 138.8, 131.8, 131.1, 129.3, 129.0, 128.1, 126.2, 105.7, 105.6, 98.6, 93.0, 69.9, 5.9 ppm.

**7-{2-[(4-Chlorophenyl)tellanyl]ethoxy}-5-hydroxy-2-phenyl-4*H*-chromen-4-one (9)** [27] Yellow solid (m.p. 140.3–143 °C; 297 mg, 0.57 mmol, isolated yield 57% purified by silica gel chromatography; eluent hexane/AcOEt 20:80). ^1^H NMR (300 MHz, CDCl_3_): δ 12.70 (s, 1H); 7.87–7.84 (m, 2H); 7.71 (d, *J* = 8.4 Hz, 2H); 7.53–7.51 (m, 3H); 7.20 (d, *J* = 8.4 Hz, 2H); 6.64 (s, 1H); 6.38–6.37 (d, *J* = 2.4 Hz, 2H); 6.27–6.26 (d, *J* = 2.1 Hz, 2H); 4.34 (t, *J* = 7.5 Hz, 2H); 3.19 (t, *J* = 7.5 Hz, 2H) ppm. ^13^C NMR (75 MHz, CDCl_3_): δ 182.3, 164.9, 164.0, 163.9, 162.1, 157.6, 140.2, 134.8, 131.8, 131.1, 129.6, 129.0, 126.2, 108.1, 105.8, 98.5, 93.1, 69.7, 6.5 ppm.

### 3.2. SARS-CoV-2 M^pro^ Expression and Purification

M^pro^ was expressed in a pET22b plasmid transformed into BL21 (DE3) Gold *E. coli* strain. Small-scale cultures grown in LB/ampicillin (100 μg/mL) at 37 °C overnight were employed for inoculating 4 L large-scale cultures of LB/ampicillin (100 μg/mL) incubated at 37 °C until reaching OD close to 0.6 at 600 nm. Protein expression was induced with 1 mM isopropyl 1-thio-β-D-galactopyranoside (IPTG) at 18 °C for 5 h. Cells were harvested by centrifugation at 4 °C for 10 min at 10,000 rpm (Beckman Coulter Avanti J-26 XP Centrifuge) and resuspended in lysis buffer (sodium phosphate 50 mM, pH 7, sodium chloride 500 mM). Cells were lysed by sonication (Sonics Vibra-Cell Ultrasonic Liquid Processor) in ice, adding benzonase 20 U/mL (Merck-Millipore, Burlington, MA, USA) and lysozyme 0.5 mg/mL (Carbosynth, Berkshire, UK). Cell debris was removed by centrifugation at 4 °C for 30 min at 20,000 rpm, and by subsequent filtration (0.45 μm pore membrane). Affinity chromatography (ÄKTA FPLC System, GE Healthcare Life Sciences, Pittsburgh, PA, USA) using a cobalt HiTrap TALON column (GE Healthcare Life Sciences) allowed fast purification in a single chromatographic step, applying an imidazole 10–250 mM gradient. Purity was assessed by SDS-PAGE, and pure protein fractions were pooled and dialyzed to remove imidazole in a buffer (sodium phosphate 50 mM, pH 7, and sodium chloride 150 mM). Protein concentration was quantitated using an extinction coefficient of 32890 M^−1^ cm^−1^ at 280 nm. Protein identity was assessed by mass spectrometry (LC-ESI-MS/MS).

### 3.3. SARS-CoV-2 M^pro^ Proteolytic Activity Assay

A continuous assay based on Förster resonance energy transfer (FRET) to measure in vitro the catalytic activity of M^pro^ was implemented by using the substrate (Dabcyl)KTSAVLQSGFRKME(Edans)-NH_2_ (Biosyntan GmbH, Berlin, Germany) [31] The enzymatic reaction was initiated by adding substrate at 20 μM (final concentration) to the enzyme at 0.2 μM (final concentration) in a final volume of 100 μL. The reaction buffer was sodium phosphate 50 mM, pH 7, NaCl 150 mM, and DMSO 2.5%. Fluorescence emission was measured in a FluoDia T70 microplate reader (Photon Technology International, Birmingham, NJ, USA) for 20 min (excitation wavelength, 380 nm; emission wavelength, 500 nm). The initial slope of the time evolution curve of the fluorescence emission signal provided a direct quantification of the enzymatic activity. The Michaelis–Menten constant, *K_m_*, and the catalytic rate constant or turnover number, *k_cat_*, were previously estimated (*K_m_* = 11 μM and *k_cat_* = 0.040 s^−1^) [13].

### 3.4. SARS-CoV-2 M^pro^ Inhibition Assay

The in vitro inhibition potency of the compounds against M^pro^ was assessed through the estimation of the inhibition constant, *K_i_*, and the half-maximal inhibitory concentration, IC_50_, from experimental inhibition curves. Inhibition curves were obtained by measuring the enzyme activity (at fixed 0.2 μM enzyme concentration and fixed 20 μM substrate concentration) as a function of compound concentration (serial 2-fold dilution from 125 µM to 0 μM), maintaining the percentage of DMSO (2.5%) constant. The enzymatic activity was quantitated as the initial slope of the substrate fluorescence emission time evolution curve, and was plotted as a function of compound concentration. The ratio between the activity (slope) in the presence and absence of the compound provides the residual percentage of activity at a given compound concentration. Nonlinear regression analysis employing a simple inhibition model (considering inhibitor depletion due to enzyme binding) allowed us to estimate the apparent inhibition constant, *K_i_^app^*, for each compound according to Equation (1):

(1) $$\begin{array}{c} \left[EI\right]=\frac{1}{2}\left({\left[I\right]}_{T}+{\left[E\right]}_{T}+K_{i}^{app}-\sqrt{{\left({\left[I\right]}_{T}+{\left[E\right]}_{T}+K_{i}^{app}\right)}^{2}-4{\left[E\right]}_{T}{\left[I\right]}_{T}}\right)\\ \left[I\right]={\left[I\right]}_{T}-\left[EI\right]=\frac{1}{2}\left({\left[I\right]}_{T}-{\left[E\right]}_{T}-K_{i}^{app}+\sqrt{{\left({\left[I\right]}_{T}+{\left[E\right]}_{T}+K_{i}^{app}\right)}^{2}-4{\left[E\right]}_{T}{\left[I\right]}_{T}}\right)\\ \frac{v\left(\left[I\right]\right)}{v\left(\left[I\right]=0\right)}=1-\frac{\left[EI\right]}{{\left[E\right]}_{T}}=\frac{1}{1+\frac{\left[I\right]}{K_{i}^{app}}} \end{array},$$

where [*EI*] is the concentration of the enzyme–inhibitor complex, [*E*]*_T_* and [*I*]*_T_* are the total concentrations of the enzyme and the inhibitor, *K_i_^app^* is the apparent inhibition constant for the inhibitors (quercetin and derivatives), [*I*] is the concentration of the free inhibitor, and *v* is the initial slope of the enzymatic activity trace at a given (free) inhibitor concentration [*I*] (or total inhibitor concentration [*I*]*_T_*). No approximation for the free inhibitor concentration (e.g., assuming to be equal to the total inhibitor concentration) was made, thus having general validity for any total enzyme and inhibitor concentration and any value of the inhibition constant (even for tight-binding inhibitors). In addition, if the inhibitor acts through a purely competitive mechanism, the previous equation can be substituted by Equation (2):

(2) $$\frac{v\left(\left[I\right]\right)}{v\left(\left[I\right]=0\right)}=\frac{1}{1+\frac{\left[I\right]}{K_{i}^{app}}}=\frac{1}{1+\frac{\left[I\right]}{K_{i}\left(1+\frac{\left[S\right]}{K_{m}}\right)}},$$

where *K_i_* is the intrinsic (i.e., substrate concentration-independent) inhibition constant, *K_m_* is the Michaelis–Menten constant for the enzyme–substrate interaction, and [*S*] is the substrate concentration. Because *K_m_* and [*S*] are known, the intrinsic inhibition constant can be determined. Furthermore, approximating the free compound concentration by the total compound concentration and neglecting ligand depletion, the *K_i_^app^* in Equation (2) is equivalent to the IC_50_, as reported in Table 3. However, it should be emphasized that the IC_50_ is an assay-dependent inhibition potency index (among other parameters, it depends on the enzyme and substrate concentrations, as well as on the *K_m_*); thus, the intrinsic inhibition constant is a better inhibition potency index. Uncertainties have been reported as 95% profile likelihood asymmetric confidence intervals, as especially recommended for equilibrium constants (i.e., binding association/dissociation constants and inhibition constants) [32].

### 3.5. Molecular Simulations

Molecular modeling of the binding between M^pro^ and compound **2d** was carried out by using an in-house version of the docking engine AutoDock Vina 1.1.2 [33]. The protocol followed was analogous to the one that some of us already used to assess the binding to the same protein of quercetin and its glycoside derivative rutin [13,14]. In brief, M^pro^ was modeled on the basis of the crystallographic structures 6Y2E and 6Y2F [29], deposited in the Protein Data Bank (PDB), which report the protein in an unliganded form and complexed with an α-ketoamide inhibitor, respectively. Compound **2d** was modeled starting from quercetin and attaching in silico the chemical adduct. An energy minimization of its structure was carried out by using the universal force field (UFF) [34]. 

The presence of the metal in compound **2d** was taken into account by modifying the docking protocol as detailed hereafter. The ordinary version of AutoDock Vina treats selenium as sulfur; therefore an updated version was used in which the correct simulation parameters were included. The well depths of the van der Waals interactions (ε_i_ = 0.291 kcal/mol) and the atomic solvation parameter (sol_par_ = −1.1 × 10^–3^) were imported from the extended scoring function of AutoDock [35]; these terms in AutoDock Vina take part also in the evaluation of the electrostatic interactions. Furthermore, the correct covalent radius of selenium (r_cov_ = 1.16 Å) was used [36]. A blind search on the whole protein volume was performed, with an exhaustiveness 16 times larger than the default value [37].

### 3.6. Cells and Viruses

Vero cells (*Cercopithecus aethiops*; kidney epithelial; ATCC CCL-81) were cultured in Dulbecco’s MEM (Thermo Fisher Scientific, Waltham, MA, USA) supplemented with 5% fetal bovine serum (heat-inactivated; Thermo Fisher Scientific, Waltham, MA, USA) and antibiotics: penicillin (100 U/mL) and streptomycin (100 μg/mL). 

Reference SARS-CoV-2 strain 026V-03883 was kindly granted by Christian Drosten, Charité—Universitätsmedizin, Berlin, Germany, by the European Virus Archive—Global (EVAg); https://www.european-virus-archive.com/, accessed on 15 April 2021). 

All SARS-CoV-2 stocks were generated by infecting monolayers of Vero cells. The cells were incubated at 37 °C under 5% CO_2_. The virus-containing medium was collected at day 2 post-infection (p.i.), aliquoted, and stored at −80 °C. Control samples from mock-infected cells were prepared in the same manner.

Virus yields were assessed by titration on fully confluent cells in 96-well plates according to the method of Reed and Muench [38]. Plates were incubated at 37 °C, and the cytopathic effect (CPE) was scored by observation under an inverted microscope.

### 3.7. Evaluation of Viral Infection

Vero cells were seeded in culture medium on 96-well plates (TPP, Trasadingen, Switzerland) at 2 days before infection. Subconfluent cells were infected with SARS-CoV-2 viruses at 1600 50% tissue culture infectious dose (TCID_50_)/mL. Infection was performed in the presence of test compounds dissolved in DMSO (100 mM stocks) or with DMSO as a control. After 2 h of incubation at 37 °C, cells were rinsed twice in PBS, and a fresh medium with the given inhibitor or solvent was added. The infection was carried out for 2 days, and the cytopathic effect (CPE) was assessed using the inverted light microscope. Culture supernatants were collected from wells where CPE reduction was observed.

### 3.8. Isolation of Nucleic Acids, Reverse Transcription, and Quantitative PCR

A viral DNA/RNA kit (A&A Biotechnology, Gdańsk, Poland) was used for nucleic acid isolation from cell culture supernatants. RNA was isolated according to the manufacturer’s instructions. 

Viral RNA was quantified using quantitative PCR coupled with reverse transcription (RT-qPCR) (GoTaq Probe 1-Step RT-qPCR System, Promega, Poland) using a CFX96 Touch real-time PCR detection system (Bio-Rad, Munich, Germany). The reaction was carried out in the presence of the probes and primers (Fwd: CAC ATT GGC ACC CGC AAT C; Rev: GAG GAA CGA GAA GAG GCT TG; probe: 6FAM-ACT TCC TCA AGG AAC AAC ATT GCC A-BHQ-1). The heating scheme was as follows: 15 min at 45 °C and 2 min at 95 °C, followed by 40 cycles of 15 s at 95 °C and 1 min at 58 °C or 60 °C. In order to assess the copy number of the N gene, standards were prepared. The PCR product was amplified and cloned into pTZ57R/T plasmids using an InsTAclone PCR cloning kit (Thermo Scientific). The resulting plasmid was linearized, and its concentration was assessed using a NanoDrop™ 2000 spectrophotometer (Thermo Fisher Scientific, Waltham, MA, USA) and the number of copies was deducted based on the Avogadro constant. The obtained standards were serially diluted and used as an input for real-time PCR.

### 3.9. Cell Viability Assay

Cell viability was evaluated using the XTT Cell Viability Assay kit (Biological Industries, Cromwell, CT, USA) according to the manufacturer’s protocol. Vero, A549ACE2+, CRFK, HRT-18, LLC-MK2, and HSF cells were cultured on 96-well plates. Cells were incubated with ACF for 24 h at 37 °C in an atmosphere containing 5% CO_2_. After incubation, the medium was discarded and 100 µL of fresh medium was added to each well. Then, 25 µL of the activated 2,3-bis-(2-methoxy-4-nitro-5-sulphenyl)-(2H)-tetrazolium-5-carboxanilide (XTT) solution was added and samples were incubated for 2 h at 37 °C. The absorbance (λ = 450 nm) was measured using a Spectra MAX 250 spectrophotometer (Molecular Devices, San Jose, CA, USA). The obtained results were normalized to the control samples, where cell viability was set to 100%.

### 3.10. Statistical Analyses

The results are expressed as mean ± standard error of the mean (SEM). For the determination of the half-maximal inhibitory concentration (IC_50_), a dose–response curve fit using a nonlinear regression model was performed using GraphPad 8.0 software. To determine the significance of the results obtained, the Kruskal–Wallis non-parametric test was used and *p*-values < 0.05 were considered significant.

## 4. Conclusions

In this communication, we reported a new metal-free protocol for the synthesis of a small library of selenoquercetin analogues. Then, the obtained compounds were screened in vitro as SARS-CoV-2 main protease inhibitors through a recently implemented method. The most significant derivatives are endowed with a low micromolar potency which is better than that shown by quercetin, highlighting that the seleno-functionalization as well as the presence of the catechol unity improves the M^pro^ inhibitory activity. The most effective M^pro^ inhibitors were further analyzed using an in cellulo model of SARS-CoV-2 infection, identifying compound **2d** as a reliable preclinical candidate. In addition, we demonstrated for the first time that quercetin **1,** besides being a low micromolar M^pro^ inhibitor, shows antiviral activity with IC_50_ = 194 μM. The introduction of the *para*-tolyl-selenyl fragment at the quercetin C-8 position led to a 24-fold improvement of the antiviral potency (IC_50_ = 8 μM), in good qualitative agreement with the indications deriving from the M^pro^ inhibition assay. Finally, experimental and in silico investigation suggested that the inhibition occurs according to a non-covalent interaction in which the selenium atom is involved in a hydrogen bond with Gln189, helping to anchor the quercetin scaffold and blocking the catalytic dyad His41/Cys145.

## Figures and Tables

**Table 1:** Optimization of the reaction conditions.

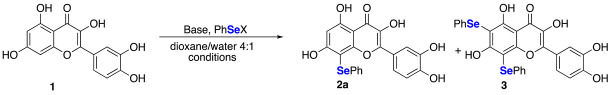						
Entry	Electrophile (**E**)	Base (**B**)	**1**:**E**:**B**	Time (h)	*T* (°C)	Yield % (**2a**:**3**)
1	PhSeCl	Tris	1:2:5	72	rt	18 (18:traces)
2	PhSeOTf ^a^	Tris	1:2:5	24	rt	11 (8:3)
3	NPSeP	Tris	1:2:5	4	rt	47 (22:25)
4	PhSeCl	Tris	1:2:5	4	80	11 (11:trace)
5	NPSeP	Tris	1:2:5	4	80	35 (10:25)
6 ^b^	PhSeCl	Tris	1:2:5	2	rt	33 (33:trace)
7 ^b^	NPSeP	Tris	1:2:5	1	rt	81 (30:51)
8 ^b^	PhSeCl	Tris	1:2:10	2	rt	22 (22:trace)
9 ^b^	PhSeCl	Et_3_N	1:2:5	2	rt	66 (55:11)
10 ^b^	PhSeCl	Arginine ^c^	1:2:5	2	rt	44 (44:trace)
11	PhSeCl	NaOH	1:2:2	6	rt	9 (9:0)

^a^ Phenylselenyl trifluoromethanesulfonate was freshly prepared from phenylselenyl bromide and silver trifluoromethanesulfonate. ^b^ The reactions were performed under an argon atmosphere. ^c^ The reaction mixture was not completely solubilized.

**Table 2:** Reaction scope.

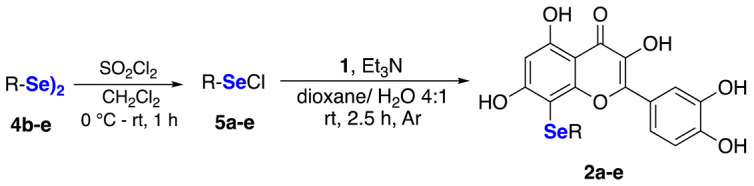		
Entry	Product	Yield (%)
1	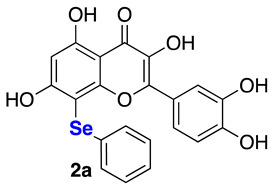	58
2	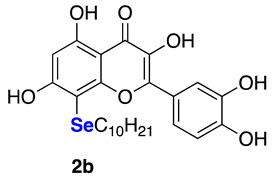	48
3	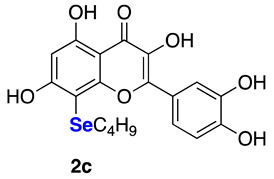	46
4	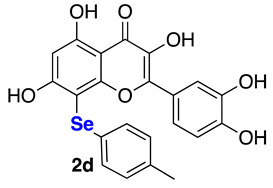	32
5	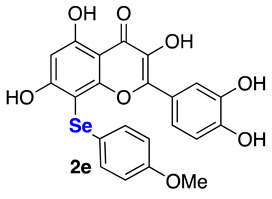	21

**Table 3:** *K*_i_ and IC_50_ values of the tested compounds ^a^.

	*K*_i_ (μM)	CI_95,Ki_	IC_50_	CI_95,IC50_
**Quercetin 1**	7.4	[5.8, 9.5]	21	[17, 28]
**2a**	1.8	[1.4, 2.2]	5.1	[4.1, 6.3]
**2b**	ND		ND	
**2c**	8.6	[6.9, 11]	24	[20, 31]
**2d**	3.8	[3.0, 5.0]	11	[8.5, 14.2]
**2e**	1.1	[0.85, 1,3]	3.0	[2.4, 3.9]
**3**	4.6	[3.7, 5.8]	13	[11, 16]
**6**	ND		ND	
**7a**	ND		ND	
**8**	1.1	[0.80, 1.6]	3.3	[2.3, 4.8]
**9**	0.77	[0.57, 1.0]	2.2	[1.7, 3.2]

^a^ Ki and IC_50_ were estimated from enzymatic assays as explained in the Materials and Methods section. ND = not determined (little or no significant inhibition at 100 μM). Uncertainties of the estimated values have been reported as 95% asymmetric confidence intervals (CI_95_).

## Data Availability

All the chemical data are within the article and its supporting information. All the biological data on antiviral activity are within the article and its supporting information.

## Supplementary Materials

The following are available online at https://www.mdpi.com/article/10.3390/ijms22137048/s1.
