# Supplementary material for: Seleno-Functionalization of Quercetin Improves the Non-Covalent Inhibition of Mpro and Its Antiviral Activity in Cells against SARS-CoV-2
Source: Int J Mol Sci. 2021 Jun 30;22(13):7048. doi: 10.3390/ijms22137048 (PMC8268238; doi:10.3390/ijms22137048)
Supplement: Supplementary file 1 [file ijms-22-07048-s001.zip › ijms-1269169-supplementary.pdf]

Supporting info

Seleno-Functionalization of Quercetin Improves the Non-covalent Inhibition of M^{pro} and Its Antiviral Activity in Cells against SARS-CoV-2

Francesca Mangiavacchi,[1] Pawel Botwina,[2,3] Elena Menichetti,[1,4] Luana Bagnoli,[1] Ornelio Rosati,[1] Francesca Marini,[1] Sérgio F. Fonseca,[5] Laura Abenante,[5] Diego Alves,[5] Agnieszka Dabrowska,[2,3] Anna Kula-Pacurar,[2] David Ortega-Alarcon,[6,7] Ana Jimenez-Alesanco,[6,7] Laura Ceballos-Laita,[6,8] Sonia Vega,[6] Bruno Rizzuti,[6,9] Olga Abian,[6,7,8,10,11] Eder J. Lenardão,[5] Adrian Velazquez-Campoy,[6,7,8,11,12] Krzysztof Pyrc,[2] Luca Sancineto,[1,*] and Claudio Santi[1,*]

[1] Group of Catalysis, Synthesis and Organic Green Chemistry, Department of Pharmaceutical Sciences, University of Perugia Via del Liceo 1, 06100 Perugia Italy; francesca.mangiavacchi@studenti.unipg.it, elena.menichetti1@studenti.unipg.it; luana.bagnoli@unipg.it, ornelio.rosati@unipg.it, francesca.marini@unipg.it.

[2] Virogenetics Laboratory of Virology, Malopolska Centre of Biotechnology, Jagiellonian University, Gronostajowa 7a, 30-387 Krakow, Poland.

[3] Microbiology Department, Faculty of Biochemistry, Biophysics and Biotechnology, Jagiellonian University, Gronostajowa 7, 30-387 Krakow, Poland.

[4] School of Science and Technology, Chemistry Division, University of Camerino, via S.Agostino , 62032 Camerino, Italy; elena.menichetti@unicam.it

[5] Laboratório de Síntese Orgânica Limpa – LASOL, CCQFA, Universidade Federal de Pelotas – UFPel, P.O. Box 354 - 96010-900, Pelotas, RS, Brazil. tec.sergio_fonseca@yahoo.com.br, laura.abenante2018@gmail.com, diego.alves@ufpel.edu.br, lenardao@ufpel.edu.br.

[6] Institute for Biocomputation and Physics of Complex Systems (BIFI), Joint Units IQFR-CSIC-BIFI, and GBsC-CSIC-BIFI, Universidad de Zaragoza, 50018 Zaragoza, Spain; dortega@bifi.es, ajimenez@bifi.es, ceballos.laita@gmail.com, svega@bifi.es, bruno.rizzuti@cnr.it, oabifra@unizar.es; adrianvc@unizar.es

[7] Departamento de Bioquímica y Biología Molecular y Celular, Universidad de Zaragoza, 50009 Zaragoza, Spain

[8] Instituto de Investigación Sanitaria de Aragón (IIS Aragon), 50009 Zaragoza, Spain

[9] CNR-NANOTEC, , SS Rende (CS), Licryl-UOS Cosenza and CEMIF.Cal, Department of Physics, University of Calabria, 87036 Rende, Italy

[10] Instituto Aragonés de Ciencias de la Salud (IACS), 50009 Zaragoza, Spain.

[11] Centro de Investigación Biomédica en Red en el Área Temática de Enfermedades Hepáticas Digestivas (CIBERehd), 28029 Madrid, Spain

[12] Fundación ARAID, Gobierno de Aragón, 50018 Zaragoza, Spain *

Copies of ^{1}H; ^{13}C and ^{77}Se spectra of compounds S3

NOESY of compound **3b** S12

Reversibility test for the binding of quercetin and compound **2d** S13

Table S1 Inhibition of the virus-caused cytopathic effect (CPE) by the compounds S14

Table S2. Inhibition of the virus-caused cytopathic effect (CPE) by the compound 1. S14

Figure S1. Antiviral activity of 2a derivative against SARS-CoV-2. S15

Figure S2. The cytotoxicity of quercetin and 2d in Vero cells. S15

Figure S3. Inhibition of the virus-caused cytopathic effect (CPE) by the quercetin and 2d compounds. S16

Figure S4. Dose-response curves of quercetin **1** and **2d** compounds determined by non-linear regression model based on RT-qPCR results S17

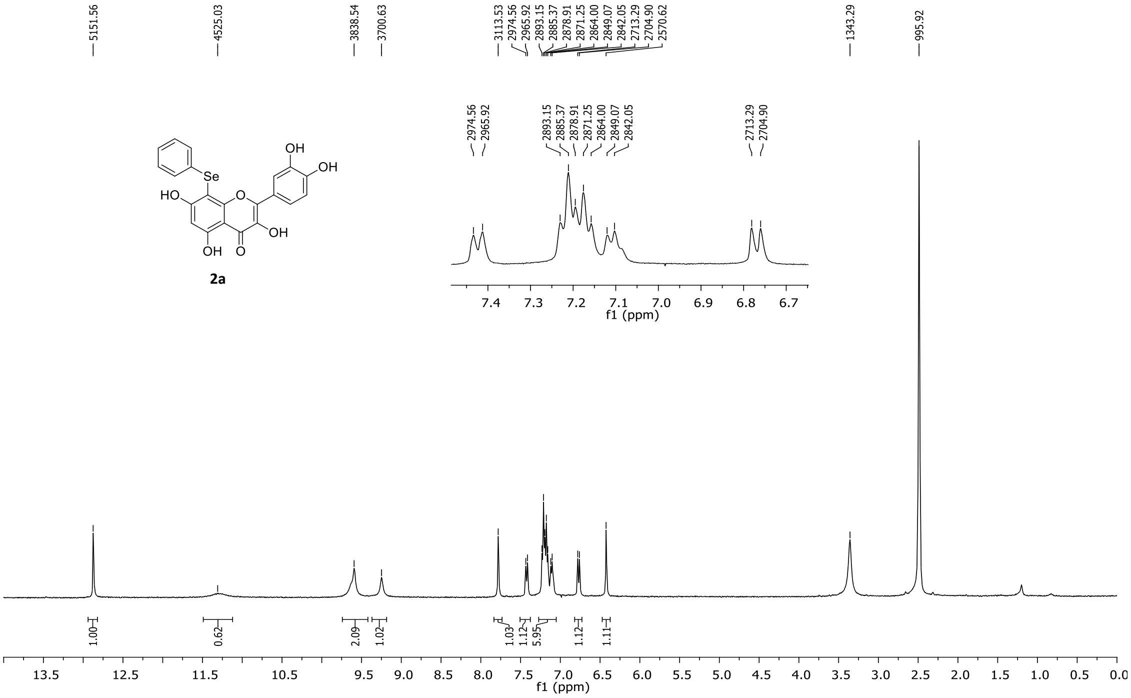
5151.56
4525.03
3838.54
3700.63
3113.53
2974.56
2965.92
2893.15
2885.37
2878.91
2871.25
2864.00
2849.07
2842.05
2713.29
2704.90
2570.62
1343.29
995.92
2a
f1 (ppm)
1.00
0.62
2.09
1.02
1.03
1.12
5.95
1.12
1.11

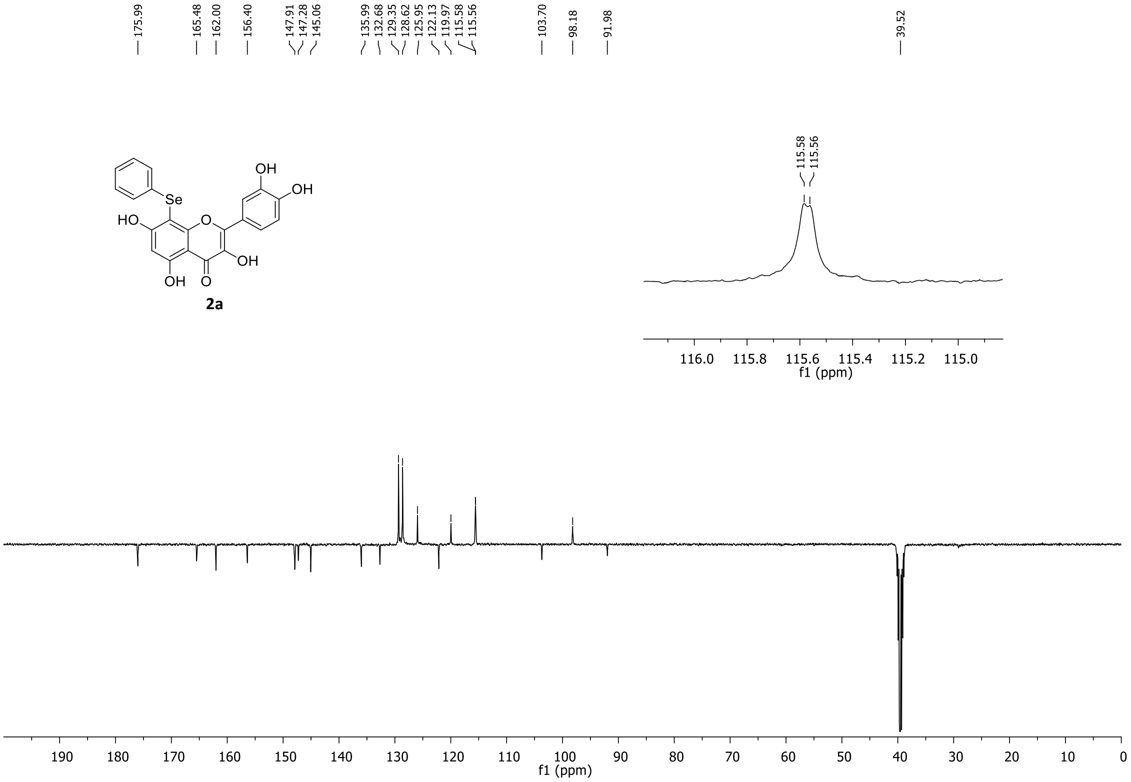
175.99
165.48
162.00
156.40
147.91
147.28
145.06
135.99
132.68
129.35
128.62
125.95
122.13
119.97
115.58
115.56
103.70
98.18
91.98
39.52
2a
f1 (ppm)

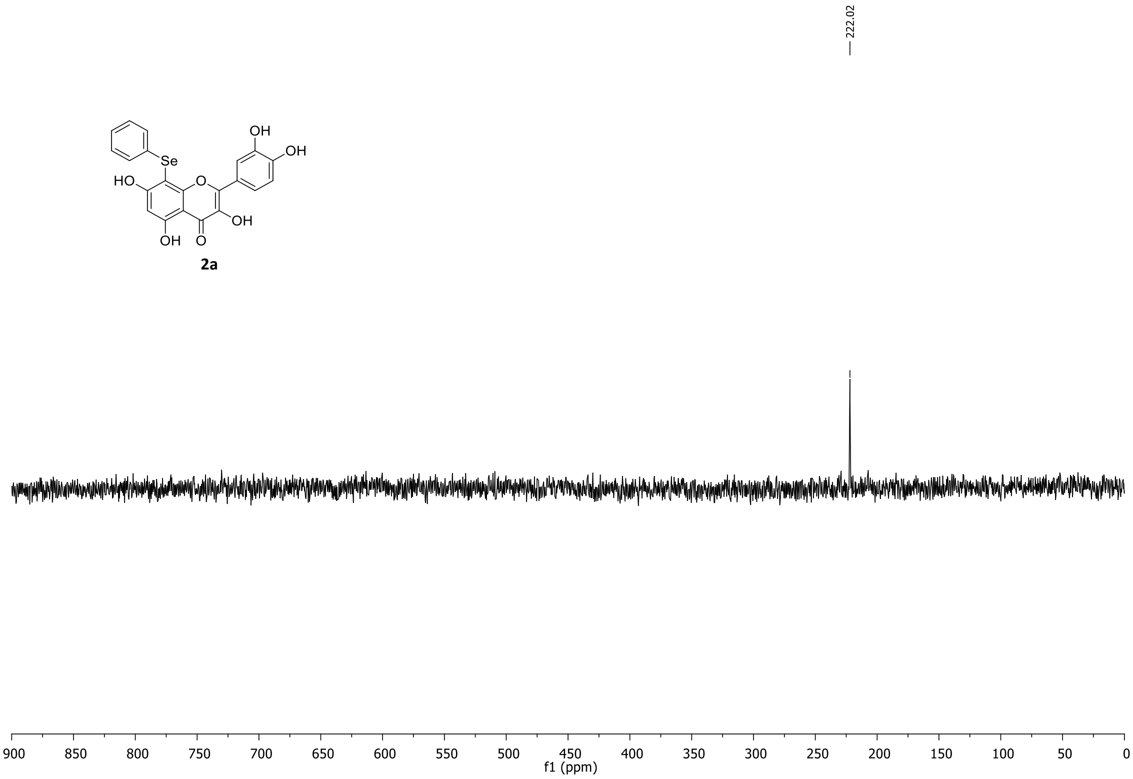
Se
HO
O
OH
OH
OH
OH
O

2a

222.02
900 850 800 750 700 650 600 550 500 450 400 350 300 250 200 150 100 50 0
f1 (ppm)

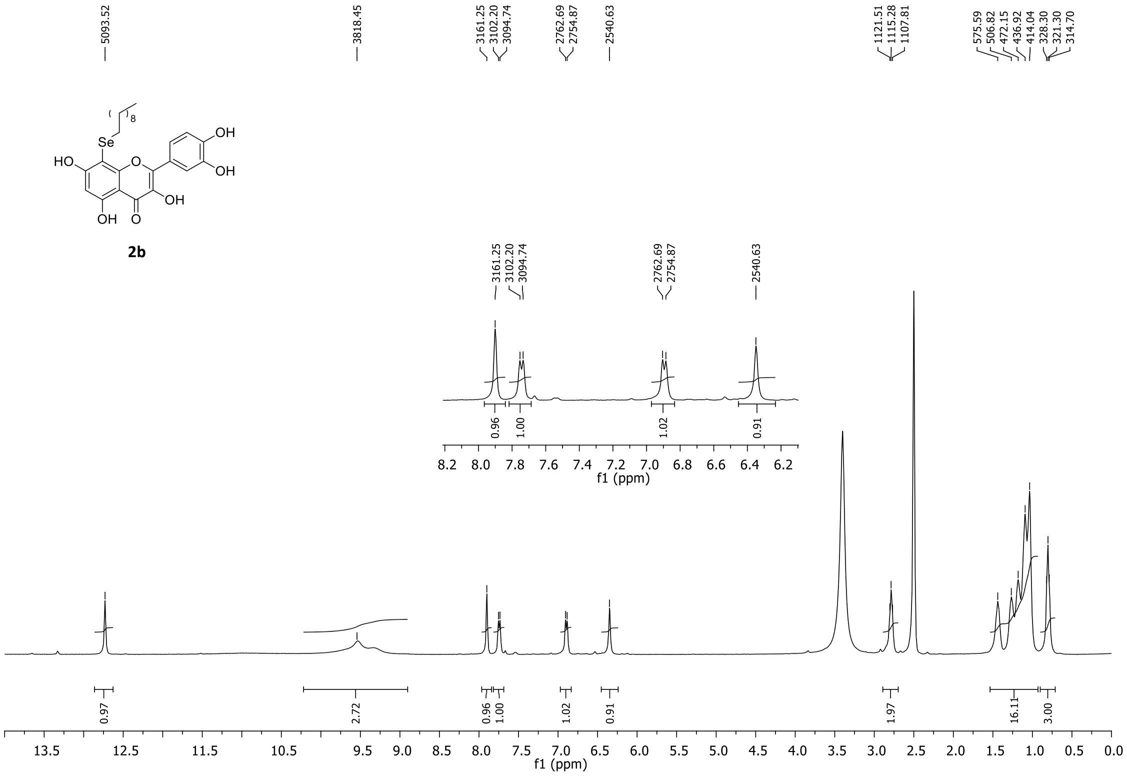
8
Se
HO
O
OH
OH
OH
OH
O

2b

5093.52
3818.45
3161.25
3102.20
3094.74
2762.69
2754.87
2540.63
1121.51
1115.28
1107.81
575.59
506.82
472.15
436.92
414.04
328.30
321.30
314.70
0.96
1.00
1.02
0.91
8.2 8.0 7.8 7.6 7.4 7.2 7.0 6.8 6.6 6.4 6.2
f1 (ppm)
0.97
2.72
1.97
16.11
3.00
13.5 12.5 11.5 10.5 9.5 9.0 8.5 8.0 7.5 7.0 6.5 6.0 5.5 5.0 4.5 4.0 3.5 3.0 2.5 2.0 1.5 1.0 0.5 0.0
f1 (ppm)

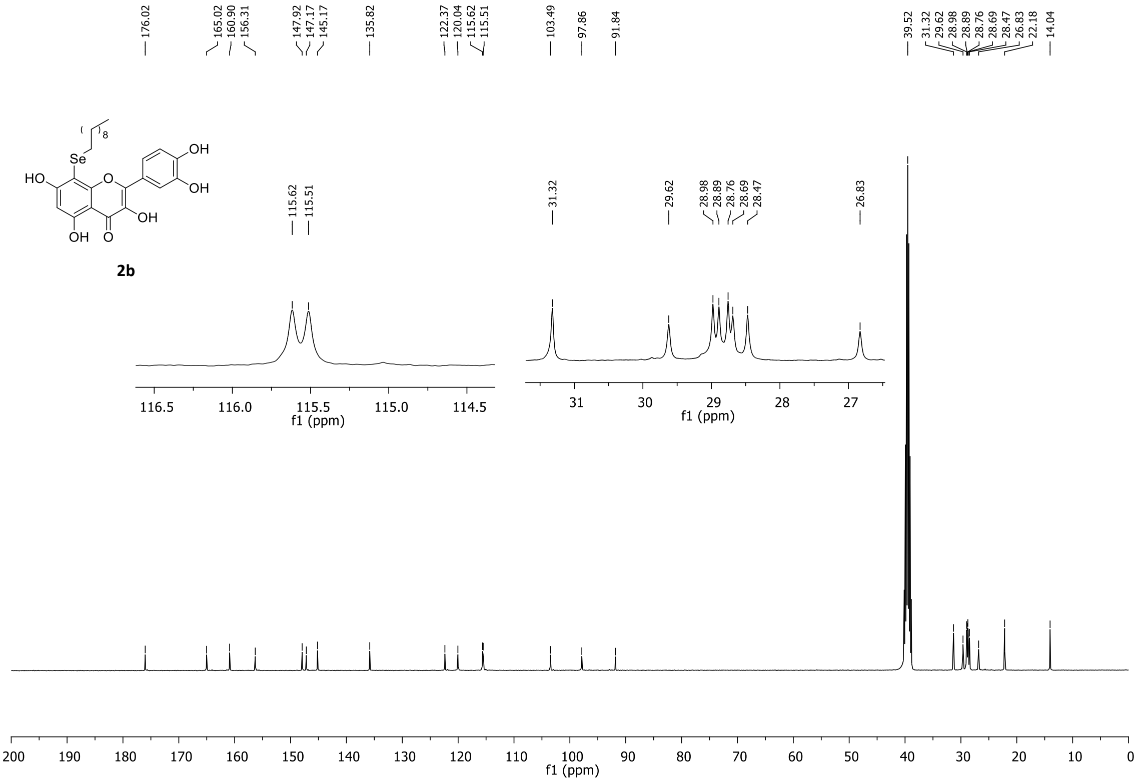
176.02
165.02
160.90
156.31
147.92
147.17
145.17
135.82
122.37
120.04
115.62
115.51
103.49
97.86
91.84
39.52
31.32
29.62
28.98
28.89
28.76
28.69
28.47
26.83
22.18
14.04
2b
f1 (ppm)

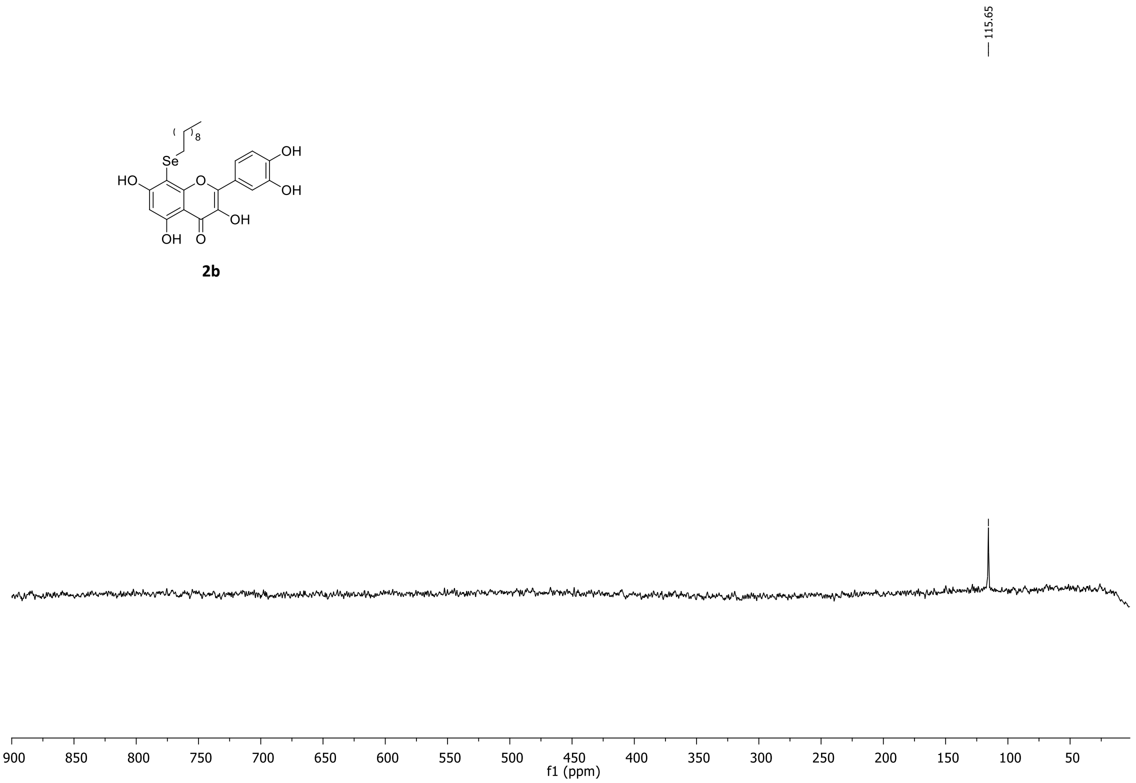
115.65
2b
f1 (ppm)

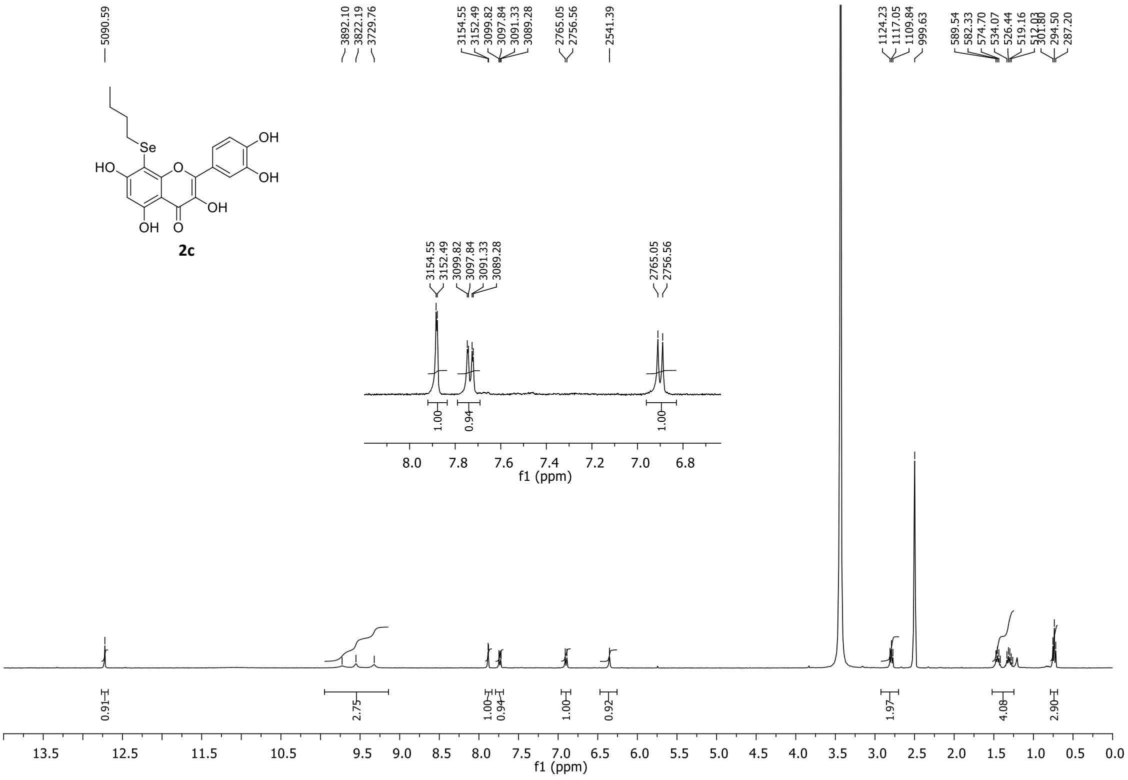
5090.59
3892.10
3822.19
3729.76
3154.55
3152.49
3099.82
3097.84
3091.33
3089.28
2765.05
2756.56
2541.39
1124.23
1117.05
1109.84
999.63
589.54
582.33
574.70
534.07
526.44
519.16
294.50
287.20
Se
HO
OH
OH
OH
OH
O
O
2c
1.00
0.94
1.00
f1 (ppm)
0.91
2.75
1.00
0.94
1.00
0.92
1.97
4.08
2.90
f1 (ppm)

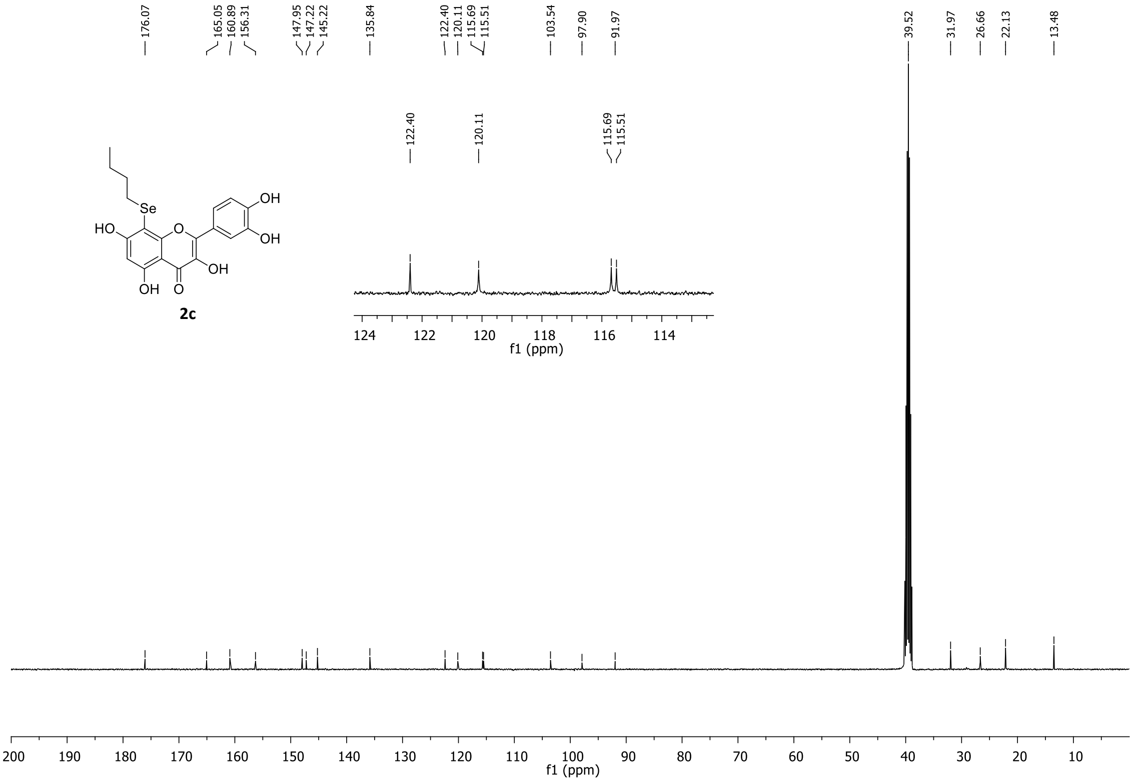
176.07
165.05
160.89
156.31
147.95
147.22
145.22
135.84
122.40
120.11
115.69
115.51
103.54
97.90
91.97
39.52
31.97
26.66
22.13
13.48
Se
HO
OH
OH
OH
OH
O
O
2c
f1 (ppm)
f1 (ppm)

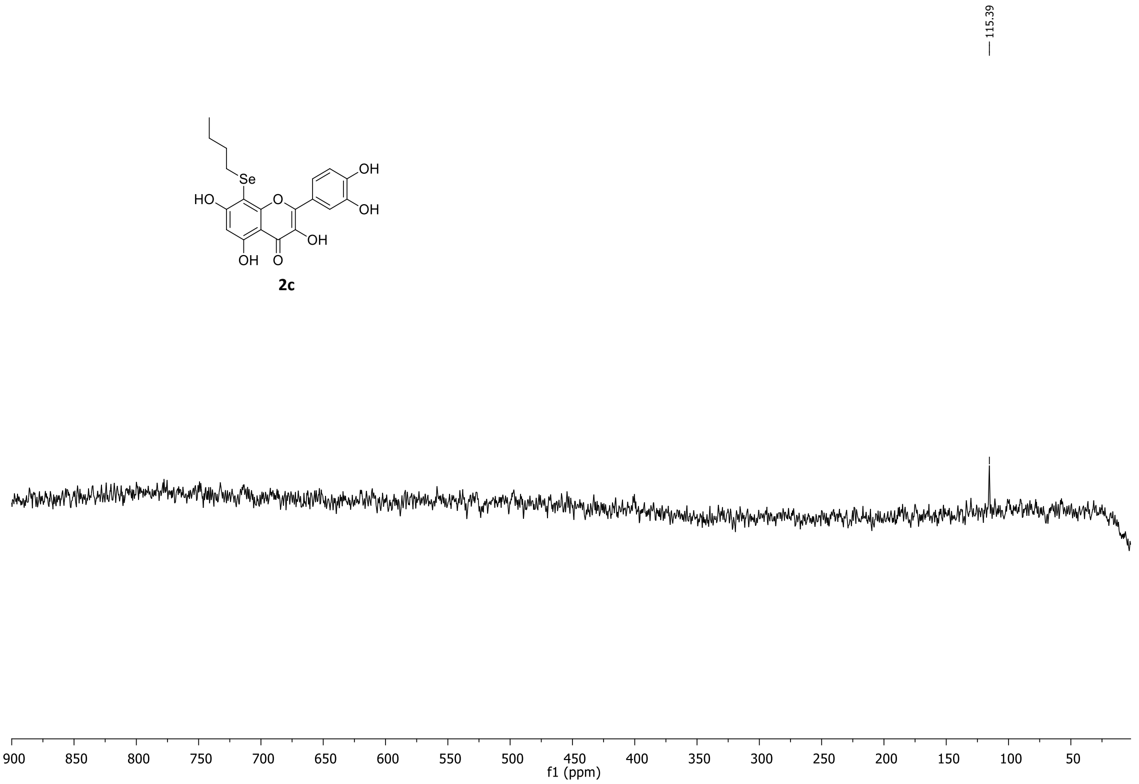
115.39
2c
Se
HO
OH
OH
OH
OH
O
O
900 850 800 750 700 650 600 550 500 450 400 350 300 250 200 150 100 50
f1 (ppm)

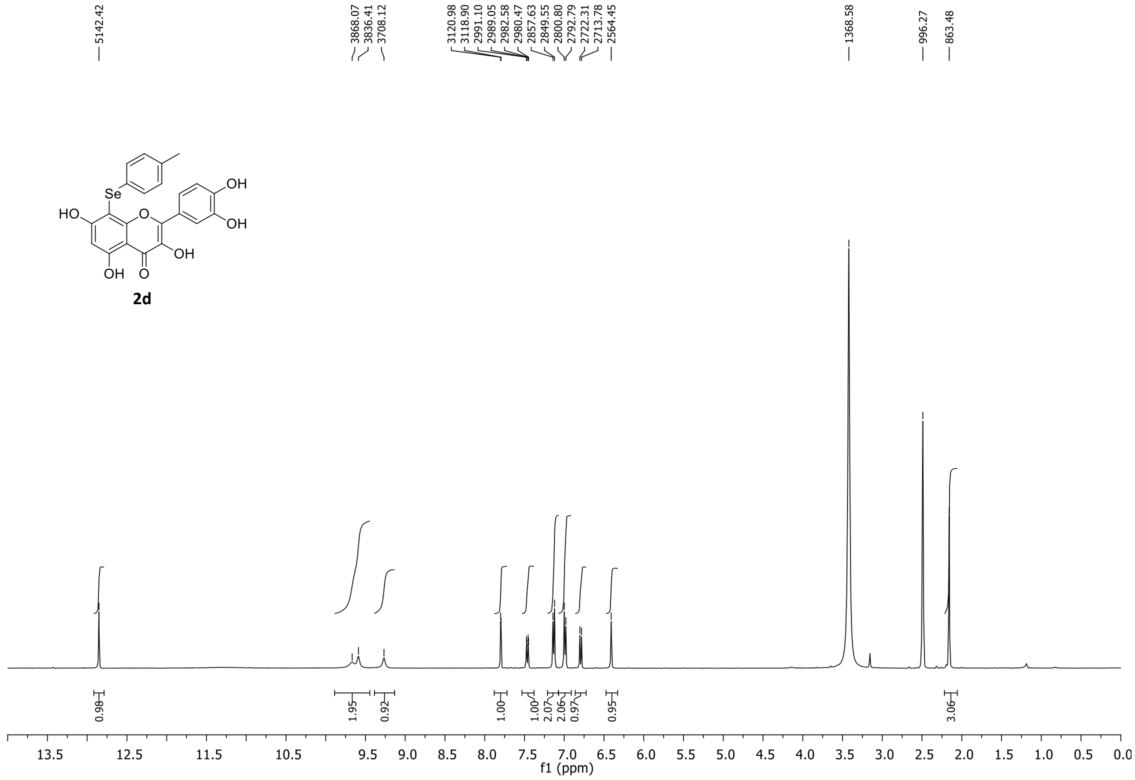
5142.42
3868.07
3836.41
3708.12
3120.98
3118.90
2991.10
2989.05
2982.58
2980.47
2857.63
2849.55
2800.80
2792.79
2722.31
2713.78
2564.45
1368.58
996.27
863.48
2d
Se
HO
OH
OH
OH
OH
O
O
0.98
1.95
0.92
1.00
1.00
2.07
2.06
0.97
0.95
3.06
13.5 12.5 11.5 10.5 9.5 9.0 8.5 8.0 7.5 7.0 6.5 6.0 5.5 5.0 4.5 4.0 3.5 3.0 2.5 2.0 1.5 1.0 0.5 0.0
f1 (ppm)

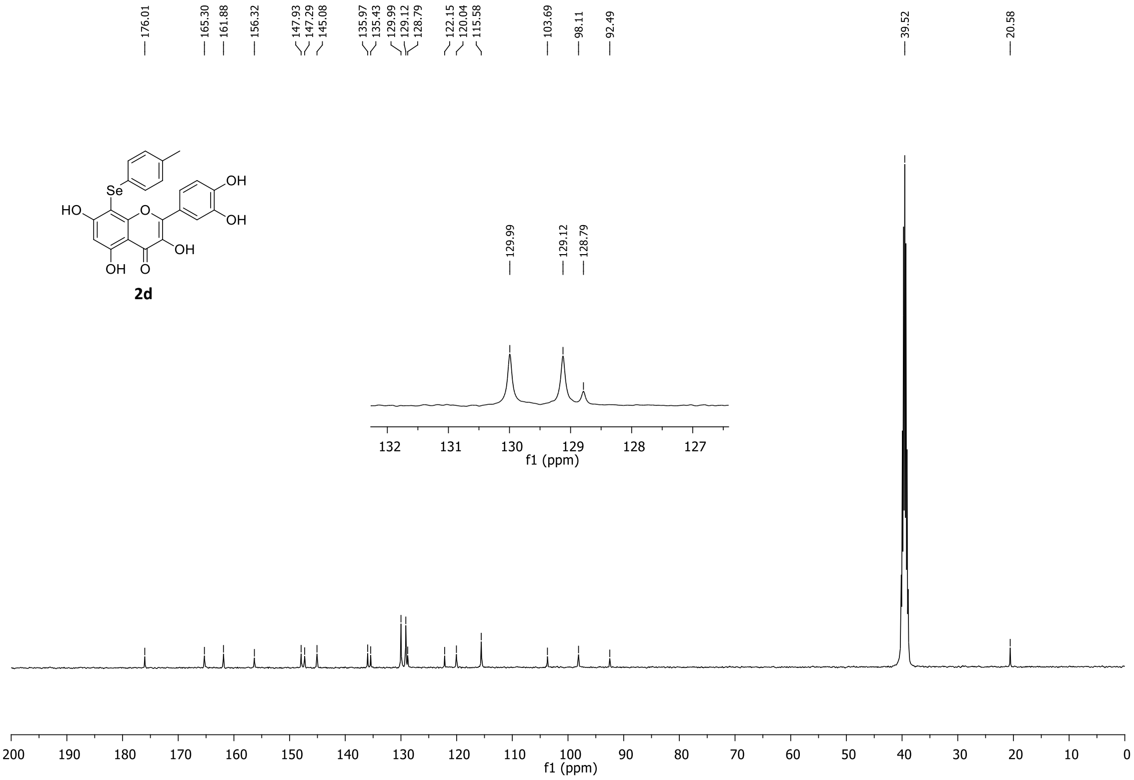
176.01
165.30
161.88
156.32
147.93
147.29
145.08
135.97
135.43
129.99
129.12
128.79
122.15
120.04
115.58
103.69
98.11
92.49
39.52
20.58
Se
HO
O
OH
OH
OH
OH
O
2d
129.99
129.12
128.79
132
131
130
129
128
127
f1 (ppm)
200
190
180
170
160
150
140
130
120
110
100
90
80
70
60
50
40
30
20
10
0
f1 (ppm)

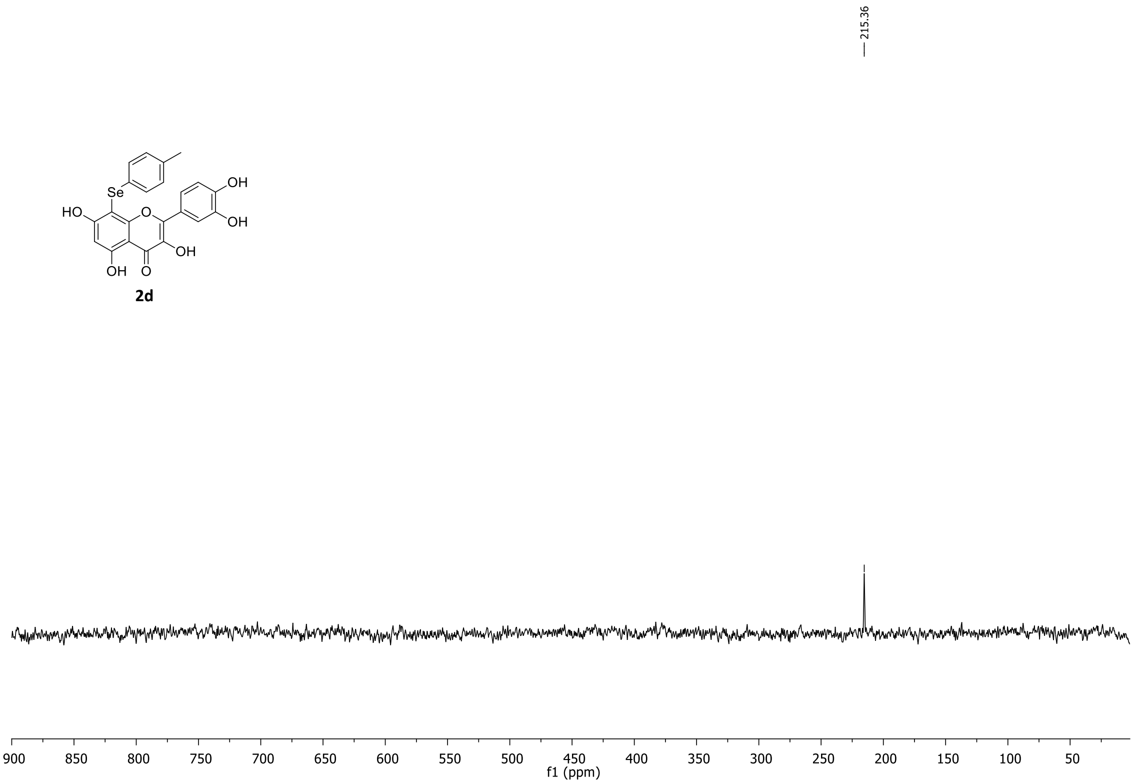
215.36
Se
HO
O
OH
OH
OH
OH
O
2d
900
850
800
750
700
650
600
550
500
450
400
350
300
250
200
150
100
50
f1 (ppm)

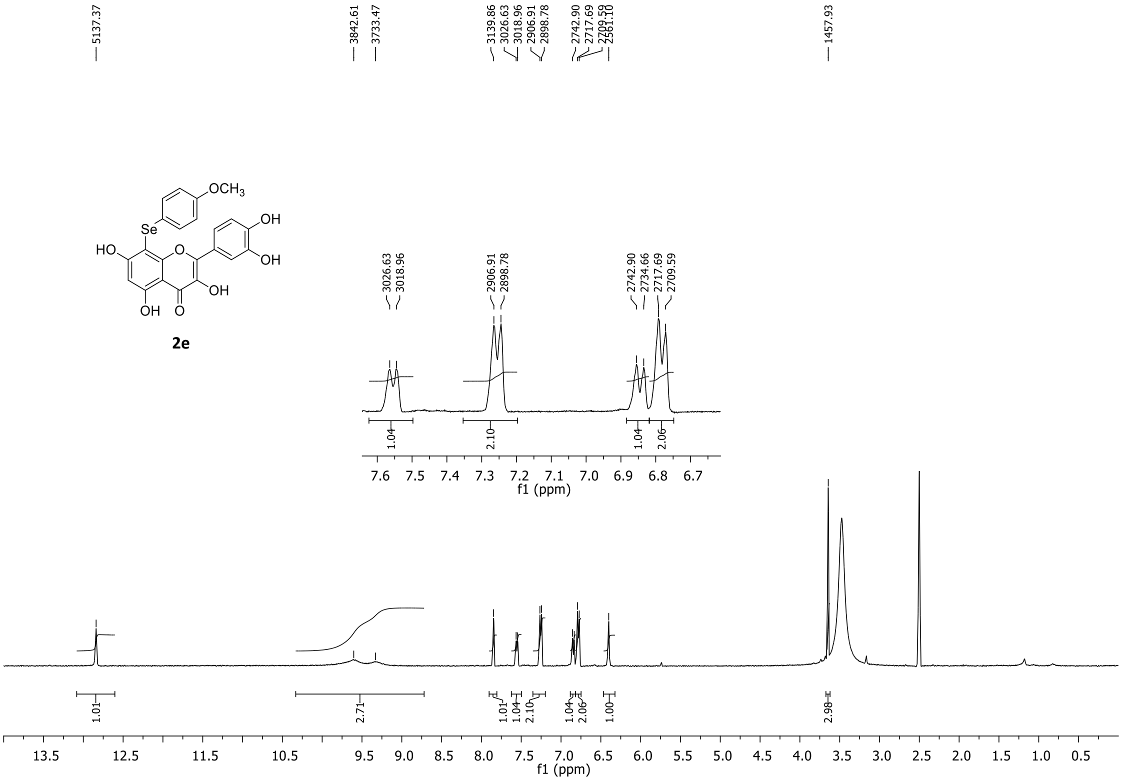

5137.37
3842.61
3733.47
3139.86
3026.63
3018.96
2906.91
2898.78
2742.90
2717.69
1457.93
3026.63
3018.96
2906.91
2898.78
2742.90
2734.66
2717.69
2709.59
1.04
2.10
1.04
2.06
7.6 7.5 7.4 7.3 7.2 7.1 7.0 6.9 6.8 6.7
f1 (ppm)
1.01
2.71
1.01
1.04
2.10
1.04
2.06
1.00
2.98
13.5 12.5 11.5 10.5 9.5 9.0 8.5 8.0 7.5 7.0 6.5 6.0 5.5 5.0 4.5 4.0 3.5 3.0 2.5 2.0 1.5 1.0 0.5
f1 (ppm)
OCH3
Se
HO
O
OH
OH
OH
OH
O

2e

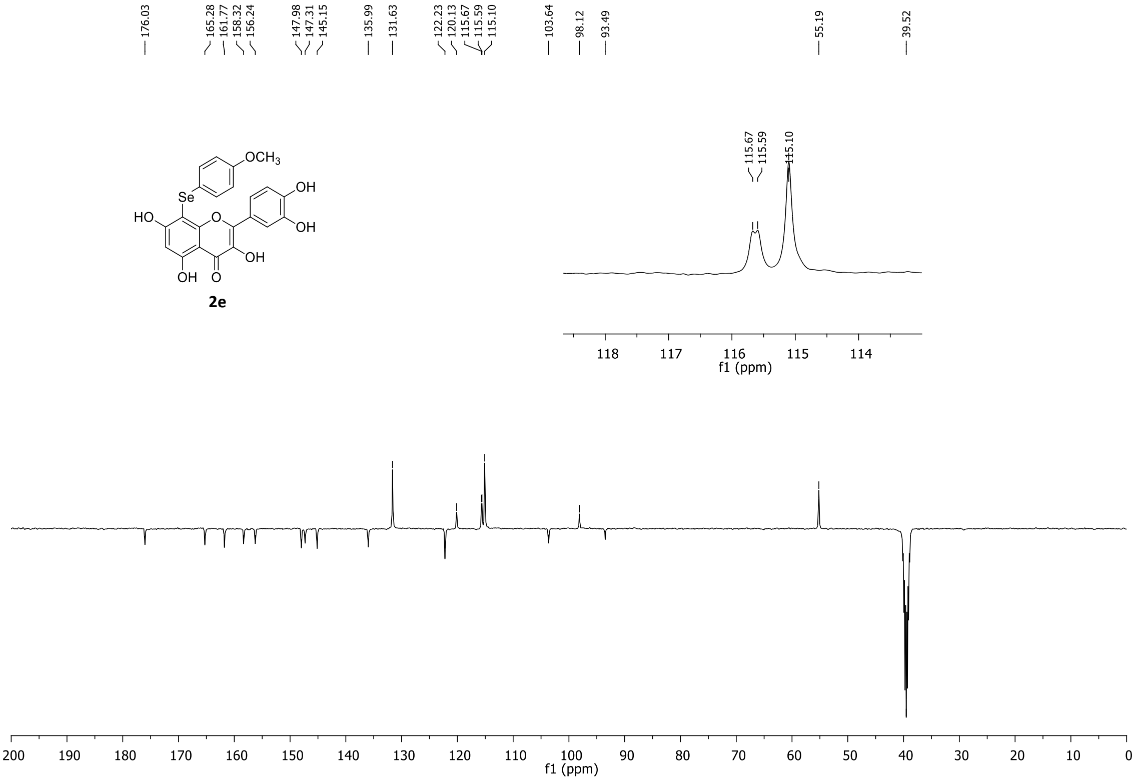

176.03
165.28
161.77
158.32
156.24
147.98
147.31
145.15
135.99
131.63
122.23
120.13
115.67
115.59
115.10
103.64
98.12
93.49
55.19
39.52
115.67
115.59
115.10
118 117 116 115 114
f1 (ppm)
200 190 180 170 160 150 140 130 120 110 100 90 80 70 60 50 40 30 20 10 0
f1 (ppm)
OCH3
Se
HO
O
OH
OH
OH
OH
O

2e

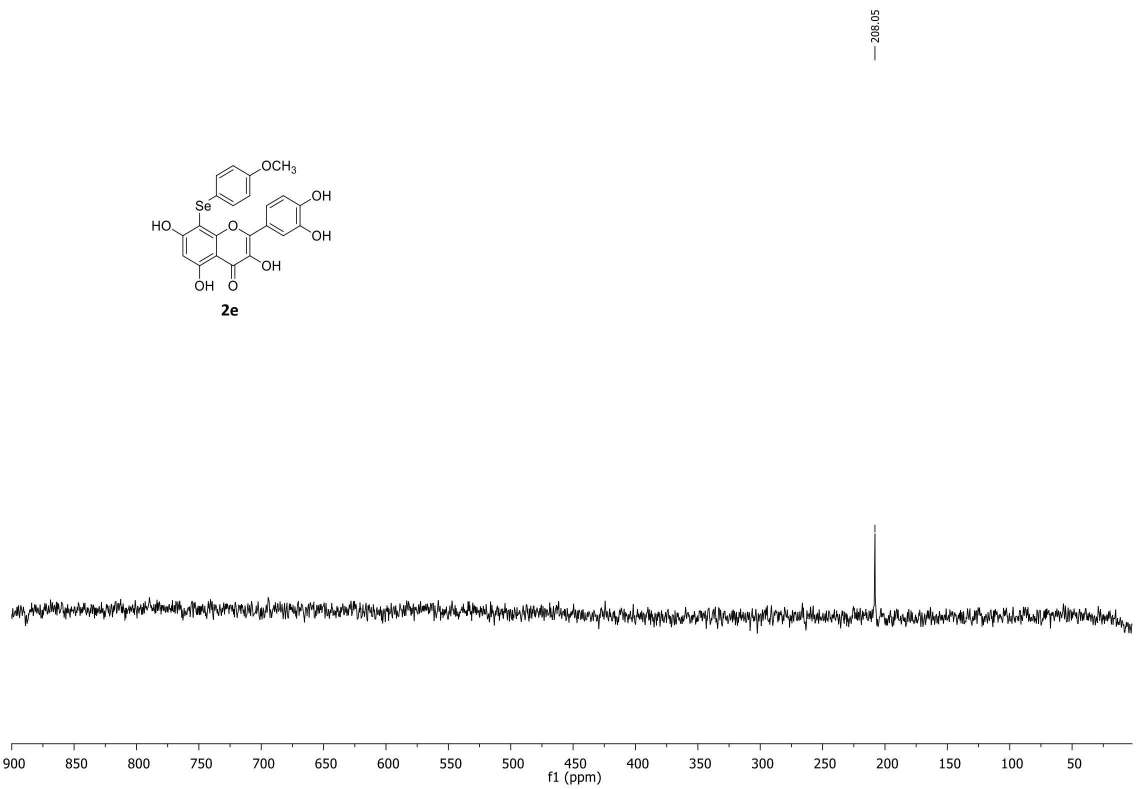
208.05
OCH3
Se
HO
OH
O
OH
OH
OH O
2e
900 850 800 750 700 650 600 550 500 450 400 350 300 250 200 150 100 50
f1 (ppm)

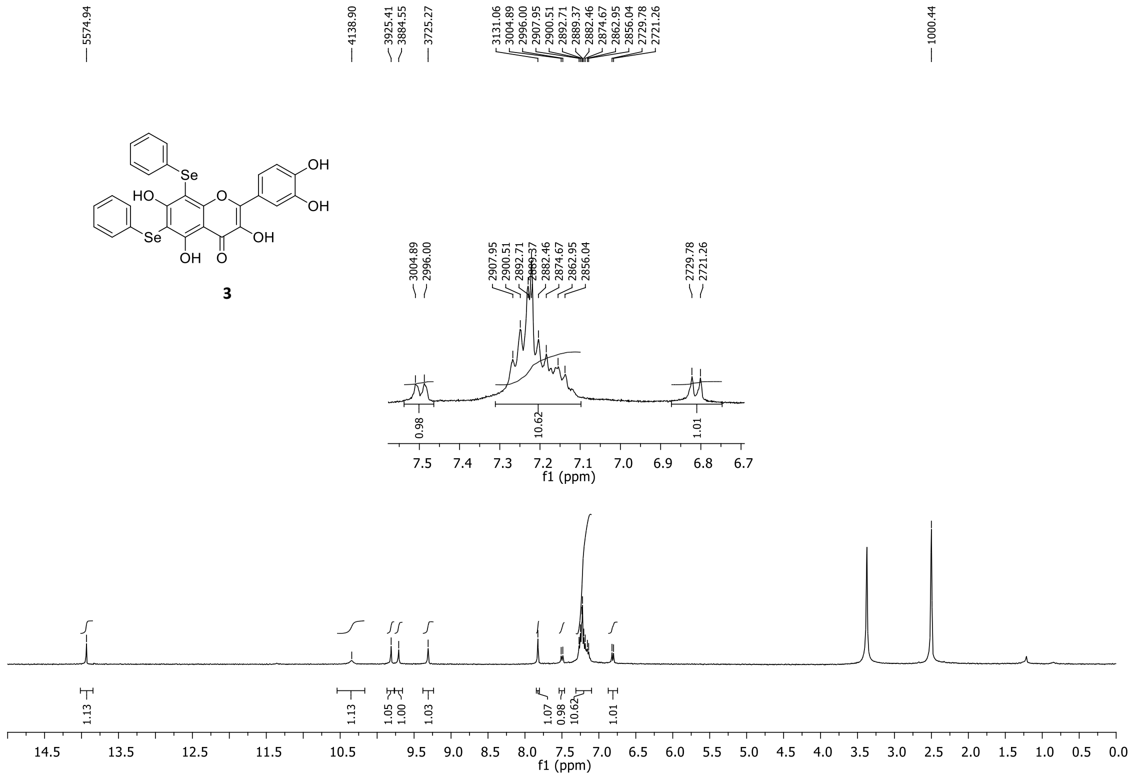
5574.94
4138.90
3925.41
3884.55
3725.27
3131.06
3004.89
2996.00
2907.95
2900.51
2892.71
2889.37
2882.46
2874.67
2862.95
2856.04
2729.78
2721.26
1000.44
Se
HO
OH
O
OH
Se
OH
OH O
3
0.98
10.62
1.01
7.5 7.4 7.3 7.2 7.1 7.0 6.9 6.8 6.7
f1 (ppm)
1.13
1.13
1.05
1.00
1.03
1.07
0.98
10.62
1.01
14.5 13.5 12.5 11.5 10.5 9.5 9.0 8.5 8.0 7.5 7.0 6.5 6.0 5.5 5.0 4.5 4.0 3.5 3.0 2.5 2.0 1.5 1.0 0.5 0.0
f1 (ppm)

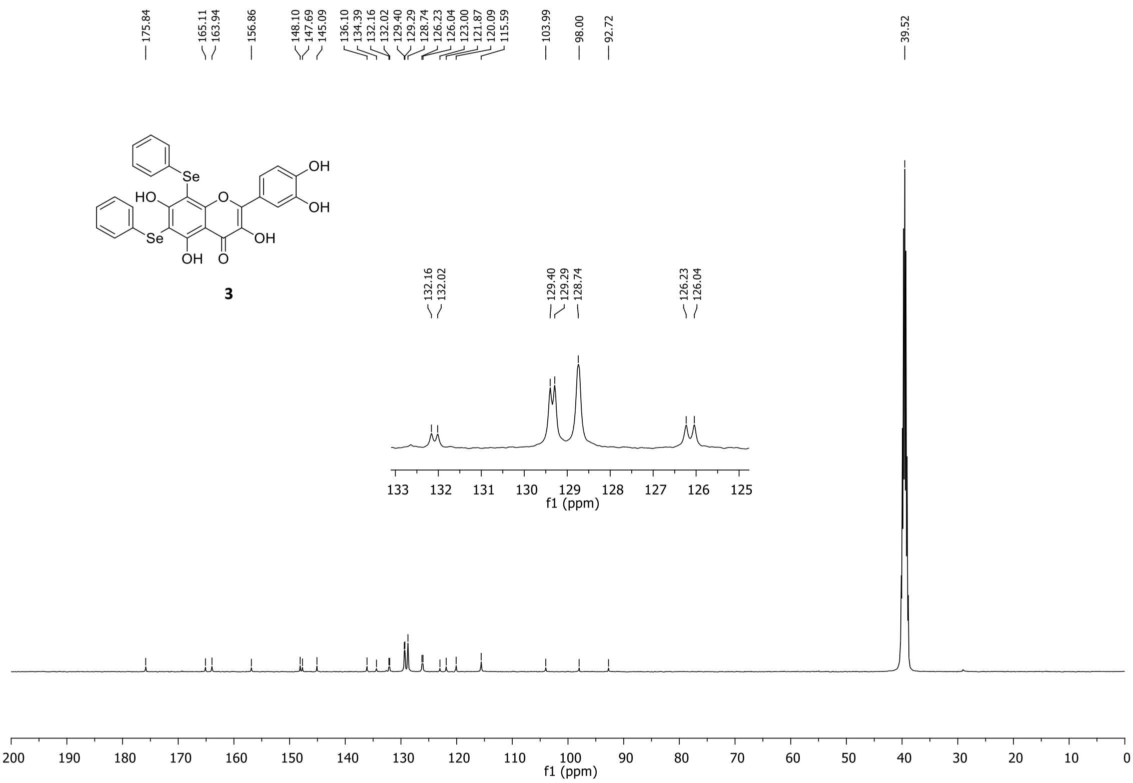
175.84
165.11
163.94
156.86
148.10
147.69
145.09
136.10
134.39
132.16
132.02
129.40
129.29
128.74
126.23
126.04
123.00
121.87
120.09
115.59
103.99
98.00
92.72
39.52
Se
HO
O
OH
OH
Se
OH
O
OH
3
f1 (ppm)

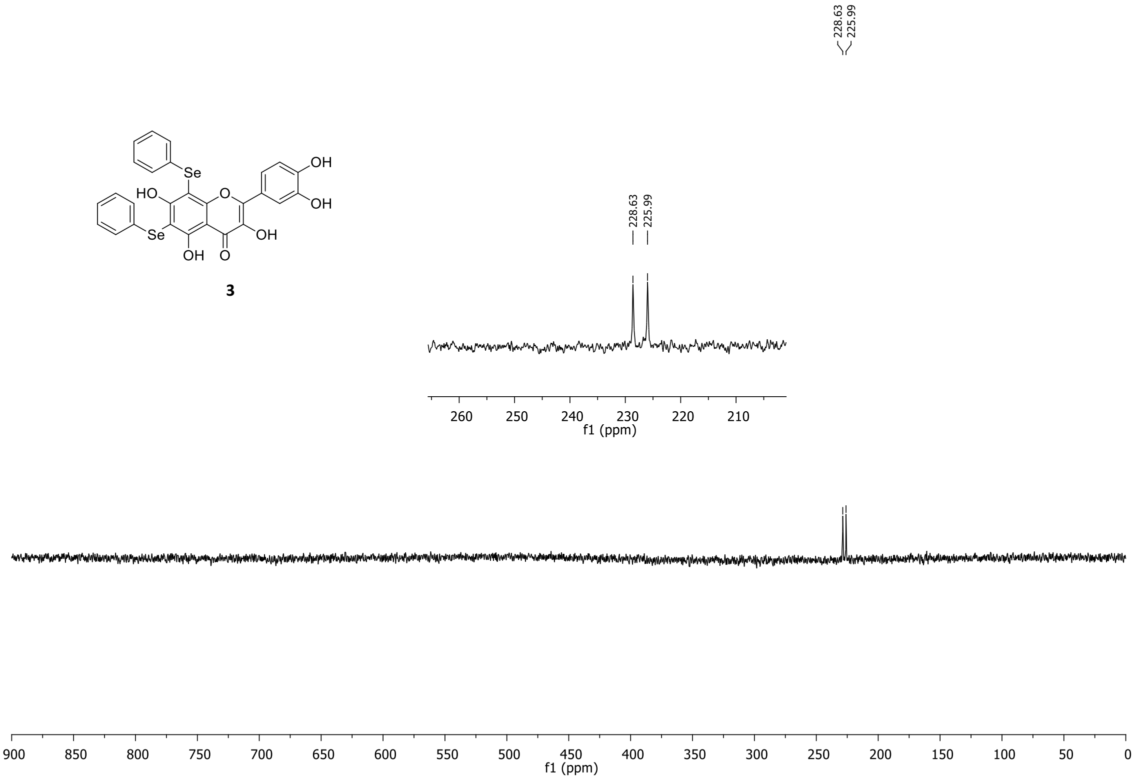
228.63
225.99
Se
HO
O
OH
OH
Se
OH
O
OH
3
f1 (ppm)

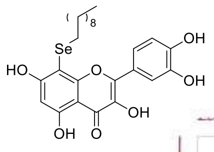

Se
HO
OH
OH
OH
OH
O
O
8

2b

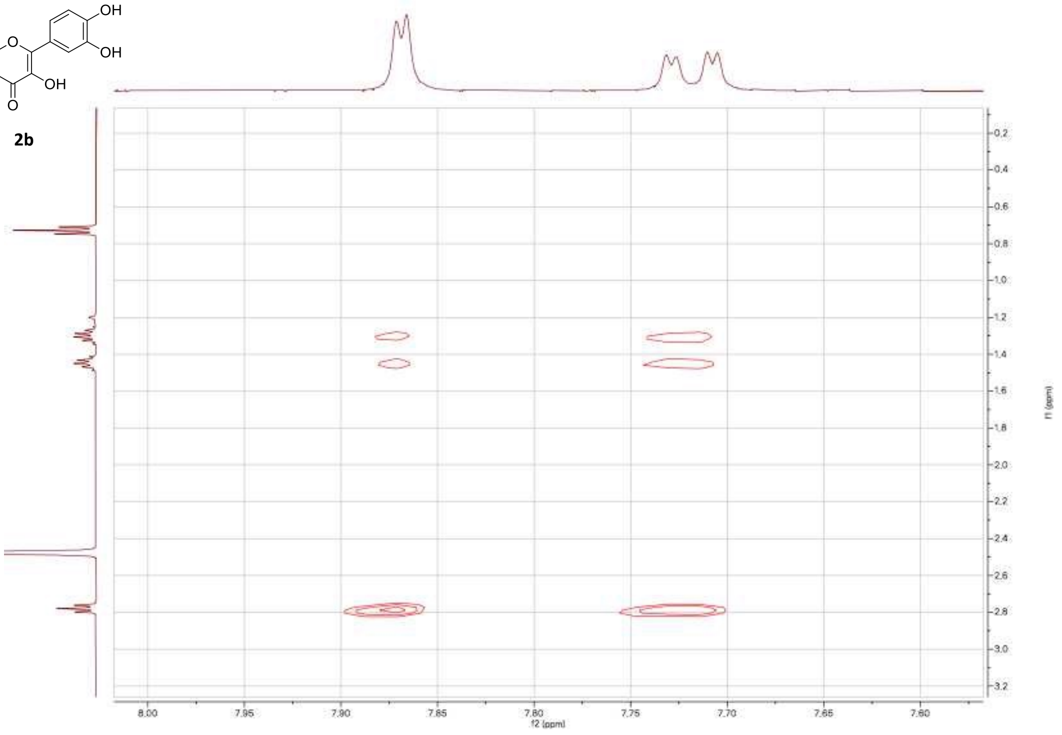

8.00
7.95
7.90
7.85
7.80
7.75
7.70
7.65
7.60
f2 (ppm)
f1 (ppm)
0.2
0.4
0.6
0.8
1.0
1.2
1.4
1.6
1.8
2.0
2.2
2.4
2.6
2.8
3.0
3.2

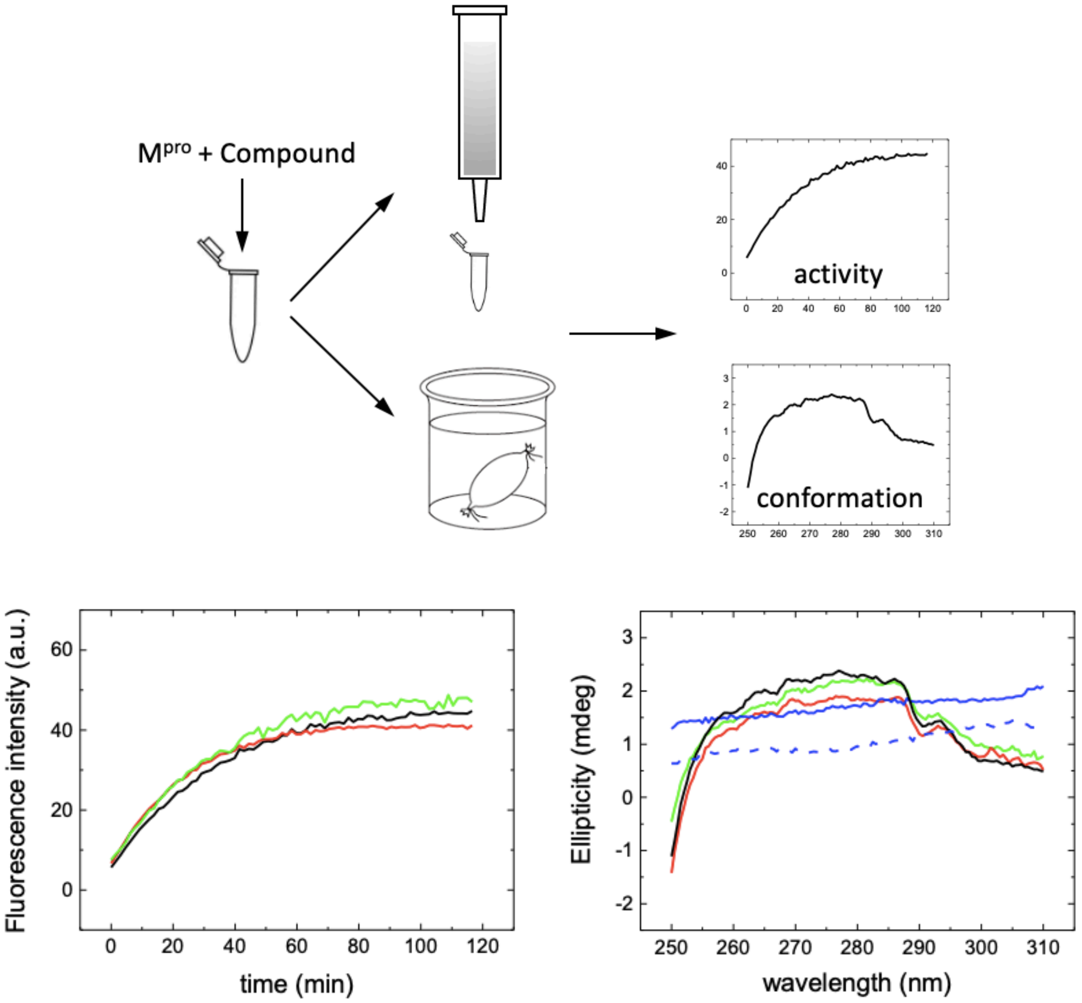

Reversibility test of M^{pro} interaction. The interaction of M^{pro} with quercetin and compound **2d** was proven to be reversible. (Upper scheme) M^{pro} (at final concentration 24 µM) was incubated with each compound (at final concentration 200 µM) for 30 min at 25 °C. Then, the sample underwent two treatments: 1) the sample was passed through a size exclusion chromatography (SEC) column and the high molecular weight fraction was isolated; and 2) the sample was dialyzed in buffer overnight. Then, the activity and the near-UV circular dichroism (CD) spectrum of M^{pro} (at the same concentration) was measured. M^{pro} with no compound was used as a control. (Left bottom plot) The activity of M^{pro} after SEC procedure: (red) after incubation with quercetin, (green) after incubation with compound **2d**, and (black) with no compound incubation. In the three cases the same specific activity was observed (within experimental error), and similar results were obtained after dialysis. (Right bottom plot) The near-UV CD spectrum of M^{pro} after SEC procedure: (red) after incubation with quercetin, (green) after incubation with compound **2d**, and (black) with no compound incubation. In the three cases the same near-UV CD spectrum was observed (within experimental error), and similar results were obtained after dialysis. The near-UV CD spectrum of M^{pro} in the presence of quercetin (dashed blue line) and in the presence of compound **2d** (solid blue line) is also shown.

Table S2 ***Inhibition of the virus-caused cytopathic effect (CPE) by the compounds.*** *(+) CPE reduction; (-) no CPE reduction; (tox) cytotoxicity observed; (prec) the compound precipitated in the medium.*

**Code**	**Concentration [µM]**				
	**100**	**75**	**50**	**25**	**10**
**Quercetin 1**	-	-	-	-	-
**2a**	tox	tox	+	-	-
**2e**	tox	tox	tox/-	-	-
**2d**	+*	+	+	+	+
**3**	tox	tox	tox/-	-	-
**8**	prec/tox	prec/tox	prec/tox/+	prec/tox/+	prec/tox/+
**9**	prec/tox	prec/-	prec-	-	-

* - morphological changes were observed in half of experiments.

Table S2. ***Inhibition of the virus-caused cytopathic effect (CPE) by the compound 1.*** *(+) CPE reduction.*

**Code**	**Concentration [µM]**				
	**500**	**400**	**300**	**250**	**200**
**Quercetin 1**	+	+	+	+	+

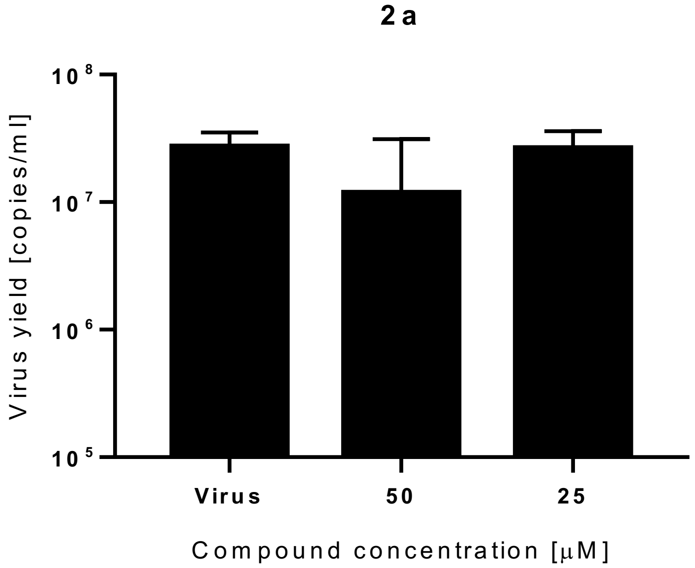

Figure S1. ***Antiviral activity of 2a derivative against SARS-CoV-2.*** *Virus replication was evaluated at given compound concentrations using RT-qPCR and the data are presented as SARS-CoV-2 RNA copies per mL of the original sample. Bars show mean value ± SEM from three independent experiments.*

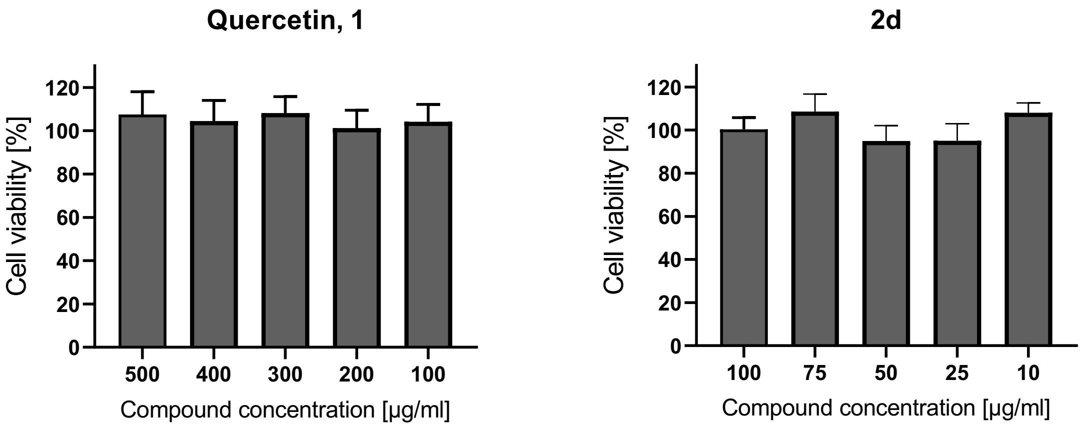

Figure S2 ***Cytotoxicity of quercetin and 2d in Vero cells****. Graphs show results of XTT assay for. All experiments were performed in triplicate. Average values with standard deviations (error bars) are presented.*

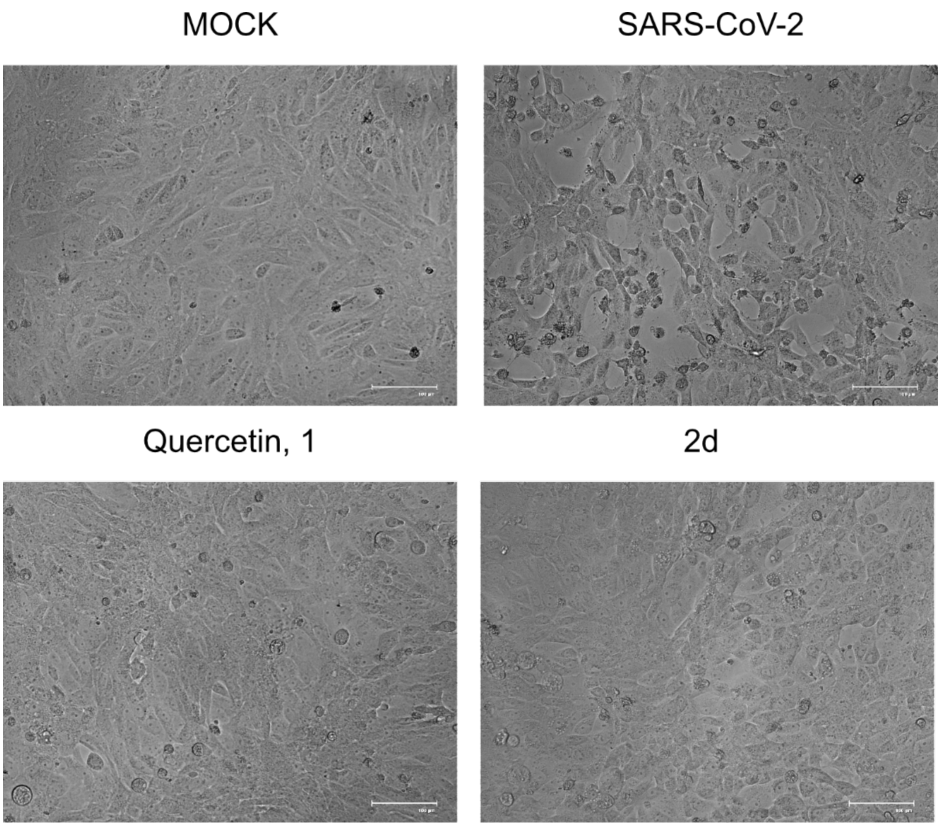

Figure S3. ***Inhibition of the virus-caused cytopathic effect (CPE) by Quercetin and 2d compounds.*** *Cytopathic effect of Vero cells infected with SARS-CoV-2 characterised by cells structural changes (cell death, cell detachment, cell circulation and appearance of vacuole) is observed under inverted light microscope (upper right panel). Reduction of CPE is shown for Quercetin and 2d compound (lower panels) at maximal non-toxic concentrations (500 and 100 µM, respectively). Negative control represents Vero cells inoculated with mock in the presence of DMSO and no CPE is observed (upper left panel). Scale bar 100 µm.*

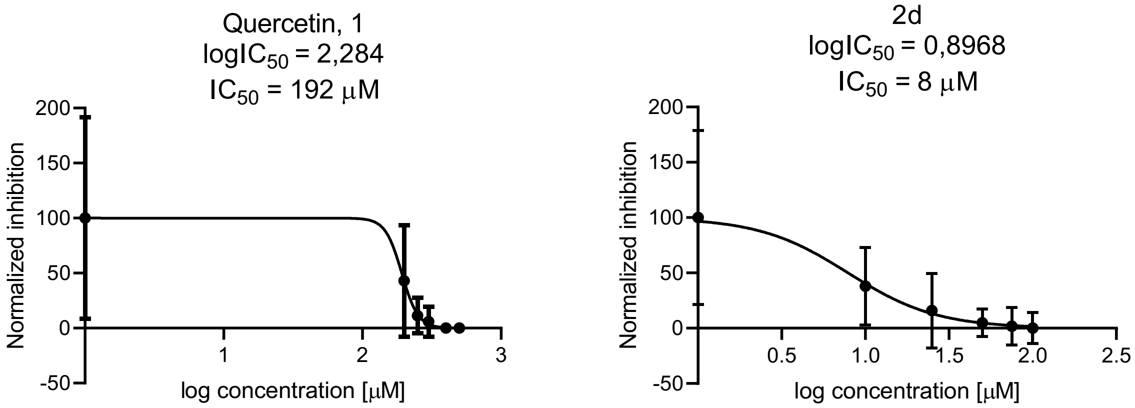

*Figure S4. Dose-response curves of quercetin **1** and **2d** compounds determined by non-linear regression model based on RT-qPCR results.*
